# Novel Strategy to Expand Super-Charged NK Cells with Significant Potential to Lyse and Differentiate Cancer Stem Cells: Differences in NK Expansion and Function between Healthy and Cancer Patients

**DOI:** 10.3389/fimmu.2017.00297

**Published:** 2017-04-05

**Authors:** Kawaljit Kaur, Jessica Cook, So-Hyun Park, Paytsar Topchyan, Anna Kozlowska, Nick Ohanian, Changge Fang, Ichiro Nishimura, Anahid Jewett

**Affiliations:** ^1^Division of Oral Biology and Oral Medicine, School of Dentistry, Los Angeles, CA, USA; ^2^Department of Tumor Immunology, Chair of Medical Biotechnology, Poznan University of Medical Sciences, Poznan, Poland; ^3^Pingan Advanced Personalized Diagnostics, Biomed Co. (USA and Beijing), Beijing, China; ^4^The Jane and Jerry Weintraub Center for Reconstructive Biotechnology, UCLA School of Dentistry, Los Angeles, CA, USA; ^5^Division of Advanced Prosthodontics, UCLA School of Dentistry, Los Angeles, CA, USA; ^6^The Jonsson Comprehensive Cancer Center, UCLA, Los Angeles, CA, USA

**Keywords:** natural killer cells, osteoclasts, Hu-BLT mice, osteoclast-expanded natural killer cells, sAJ2

## Abstract

Natural killer (NK) cells are known to target cancer stem cells and undifferentiated tumors. In this paper, we provide a novel strategy for expanding large numbers of super-charged NK cells with significant potential to lyse and differentiate cancer stem cells and demonstrate the differences in the dynamics of NK cell expansion between healthy donors and cancer patients. Decline in cytotoxicity and lower interferon (IFN)-γ secretion by osteoclast (OC)-expanded NK cells from cancer patients correlates with faster expansion of residual contaminating T cells within purified NK cells, whereas healthy donors’ OCs continue expanding super-charged NK cells while limiting T cell expansion for up to 60 days. Similar to patient NK cells, NK cells from tumor-bearing BLT-humanized mice promote faster expansion of residual T cells resulting in decreased numbers and function of NK cells, whereas NK cells from mice with no tumor continue expanding NK cells and retain their cytotoxicity. In addition, dendritic cells (DCs) in contrast to OCs are found to promote faster expansion of residual T cells within purified NK cells resulting in the decline in NK cell numbers from healthy individuals. Addition of anti-CD3 mAb inhibits T cell proliferation while enhancing NK cell expansion; however, expanding NK cells have lower cytotoxicity but higher secretion of IFN-γ. Expansion and functional activation of super-charged NK cells by OCs is dependent on interleukin (IL)-12 and IL-15. Thus, in this report, we not only provide a novel strategy to expand super-charged NK cells, but also demonstrate that rapid and sustained expansion of residual T cells within the purified NK cells during expansion with DCs or OCs could be a potential mechanism by which the numbers and function of NK cells decline in cancer patients and in BLT-humanized mice.

## Statement of Translational Relevance

Osteoclast (OC)-induced expansion of highly purified cancer patients’ natural killer (NK) cells and those from tumor-bearing-humanized mice are limited because of the outgrowth of the small population of contaminating T cells. These results may provide in part an explanation for why NK cells decrease in number in cancer patients. Moreover, we show that dendritic cells (DCs) expand T cells, whereas OCs expand NK cells suggesting microenvironmental differences for the expansion of T and NK cells. In addition, we designed a strategy to expand large numbers of super-charged NK cells for use in immunotherapeutic strategies to eliminate cancer stem cells and control tumor growth by differentiation of stem-like/poorly differentiated tumors. Our paper has a significant translational focus, which should facilitate future cancer immunotherapies.

## Introduction

Natural killer cells lyse and differentiate cancer stem cells/undifferentiated tumors with lower expression of major histocompatibility complex (MHC) class I, CD54, B7H1, and higher expression of CD44 (1, 2). Medium and high cytotoxic activity of peripheral-blood lymphocytes are associated with reduced cancer risk, and high NK-cell infiltration of the tumor is associated with a better prognosis (3, 4), whereas low activity is associated with increased cancer risk (5).

Suppression of NK cells is mediated by downregulation of NK receptors in the tumor microenvironment (6–13). Function of NK cells was shown previously to be significantly reduced in tumor patients (5, 6, 8–12, 14–16).

Several *in vitro* NK expansion techniques have been developed to allow for a higher therapeutic cell dose (Table S1 in Supplementary Material) (17–25). The stimulation of peripheral blood mononuclear cells (PBMCs) or purified population of NK cells with feeder cells such as K562 cells expressing interleukin (IL)-15 and 41BB ligand, EBV-TM-LCL, Wilm’s tumor, or irradiated PBMCs have resulted in greater numbers of NK cells with adequate function (Table S1 in Supplementary Material) (23, 26, 27). The generated NK cells expressed higher levels of NKG2D, natural cytotoxicity receptors, DNAM-1, and ICAM-1 (25). Thus, various methods to obtain *ex vivo* expanded, activated, and CD3^+^ T cell-depleted NK cells have been established for clinical use (28). In addition, Miller and colleagues established the safety and efficacy of adoptive cellular transfer of human leukocyte antigen-haploidentical NK cells in patients with advanced cancer (29). Additionally, clinical trials have shown that allogeneic NK cells play a therapeutic role in solid tumors and are safe for transfer into patients (30–32).

Immunotherapy with NK cells has been limited due to inability to obtain sufficient numbers of highly functional NK cells. In this paper, we provide a novel strategy to expand highly functional NK cells using OCs as feeder cells, at the levels which are significantly superior to those established by other methodologies (Table S1 in Supplementary Material). In addition, expansion of patients’ NK cells unlike NK cells from healthy individuals is significantly limited due to the expansion of a small fraction of contaminating T cells which could potentially crowd out or inhibit NK cell expansion by their faster proliferating capability. This trend was also seen in NK cell cultures from tumor-bearing-humanized mice.

## Materials and Methods

### Cell Lines, Reagents, and Antibodies

RPMI 1640 complete medium with 10% fetal bovine serum (FBS) (Gemini Bio-Product) was used for cell cultures. Oral squamous carcinoma cells and oral squamous carcinoma stem-like cells (OSCSCs) were isolated from cancer patients with tongue tumors at UCLA (2, 33–35). Alpha-MEM (Life Technologies, CA, USA) with 10% FBS was used for OCs and DCs cultures. M-CSF was purchased from Biolegend (CA, USA) and RANKL, GM-CSF, and IL-4 were purchased from PeproTech (NJ, USA) and rh-IL-2 was obtained from NIH-BRB. Human CD3/CD28 T cell activator was purchased from Stem Cell Technologies.

Antibodies for MHC-I, KIR2, KIR3, CD44, CD54, B7H1, CD16, NKG2D, MICA/B, KLGR1, CD45, CD3/16/56, CD8, CD3, CD4, GL3, NKp40, NKp30, NKp44, NKp46, and CD94 were purchased from Biolegend (San Diego, CA, USA). ULBP 1–6 antibodies were purchased from R&D Systems. Propidium iodide (PI) was purchased from Sigma (St. Louis, MO, USA). sAJ2 was prepared as described previously (35).

### Purification of NK Cells and T Cells from Human PBMCs and hu-BLT Splenocytes

Natural killer cells and T cells were purified as described previously (36). T cells from hu-BLT splenocytes were positively purified using isolation kits from Stem Cell Technologies (Stem Cell Technologies, Vancouver, BC, Canada).

### Purification of Monocytes and Generation of OCs from hu-BLT Mice and OCs and DCs from Human PBMCs

The study as well as the procedures were approved by the UCLA Institutional Review Board (IRB), and all participants signed written informed consent in accordance with the Declaration of Helsinki. Human monocytes were purified as described previously (36). Monocytes from hu-BLT mice were positively isolated from bone marrow using human CD14 isolation kit (eBioscience, San Diego, CA, USA). Greater than 95% purity was achieved for each subset based on flow cytometric analysis. Monocytes were differentiated to OCs by treating with M-CSF (25 ng/ml) and RANKL (25 ng/ml) for 21 days. To obtain DCs, human monocytes were treated with GM-CSF (150 ng/ml) and IL-4 (50 ng/ml) for 7 days.

### Expansion of NK Cells

Human purified and hu-BLT enriched NK cells were activated with rh-IL-2 (1,000 U/ml) and anti-CD16mAb (3 μg/ml) for 18–20 h before they were cocultured with feeder cells and sAJ2. The culture media was refreshed with rh-IL-2 every 3 days.

### Tumor Implantation and Tissue Preparation from hu-BLT Mice

Animal research described in this manuscript was performed under the written approval of the UCLA Animal Research Committee (ARC) in accordance to all federal, state, and local guidelines. Combined immunodeficient NOD.CB17-Prkdcscid/J and NOD.Cg-Prkdcscid Il2rgtm1Wjl/SzJ (NSG lacking T, B, and NK cells) were purchased from Jackson Laboratory. Humanized-BLT (hu-BLT; human bone marrow/liver/thymus) mice were prepared on NSG background as described previously (37, 38). To establish orthotopic tumors, mice were first anesthetized with isoflurane in combination with oxygen, and tumor cells were directly injected in the floor of mouth in suspension with 10 μl HC Matrigel (Corning, NY, USA) (1 × 10^6^ cells). Four to five weeks after the tumor injections, mice were euthanized and bone marrow, spleen, and blood were harvested and single cell suspensions were prepared (39).

### ELISA and Multiplex Cytokine Array Kit

Single ELISAs and multiplex assays were performed as described previously (36).

### Cancer Stem Cell Differentiation with NK Cell Supernatants

Supernatants from NK cells were prepared and used for differentiation of OSCSCs as described previously (35). Day 13 supernatants from OC-expanded NK cells were used for differentiation.

### Surface Staining and Cell Death Assays

Staining was performed by labeling the cells with antibodies or PI as described previously (36, 40, 41). Flow cytometry analysis was performed using Beckman Coulter Epics XL cytometer (Brea, CA, USA) and results were analyzed using FlowJo vX software (Ashland, OR, USA).

### ^51^Cr Release Cytotoxicity Assay

The ^51^Cr release assay was performed as described previously (42).

### Statistical Analysis

An unpaired, two-tailed Student’s *t*-test or one way ANOVA were performed to compare different groups depending on the experimental design. The *p*-values were expressed within the figures as follows: ***p-value < 0.001, **p-value: 0.001–0.01, *p-value: 0.01–0.05.

## Results

### Preferential Expansion of NK or T Cells by OCs or DCs, Respectively

**Figure 1:**
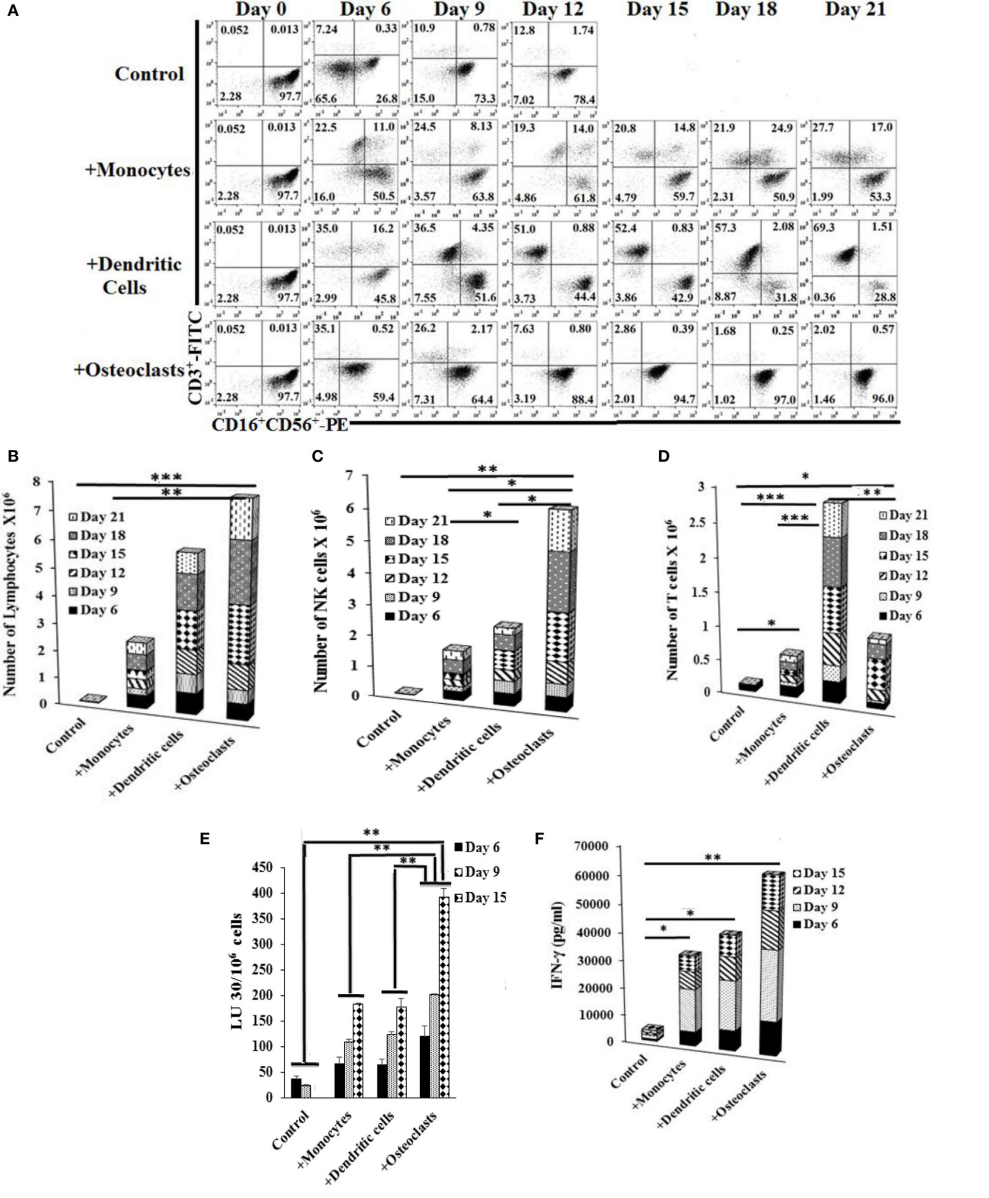

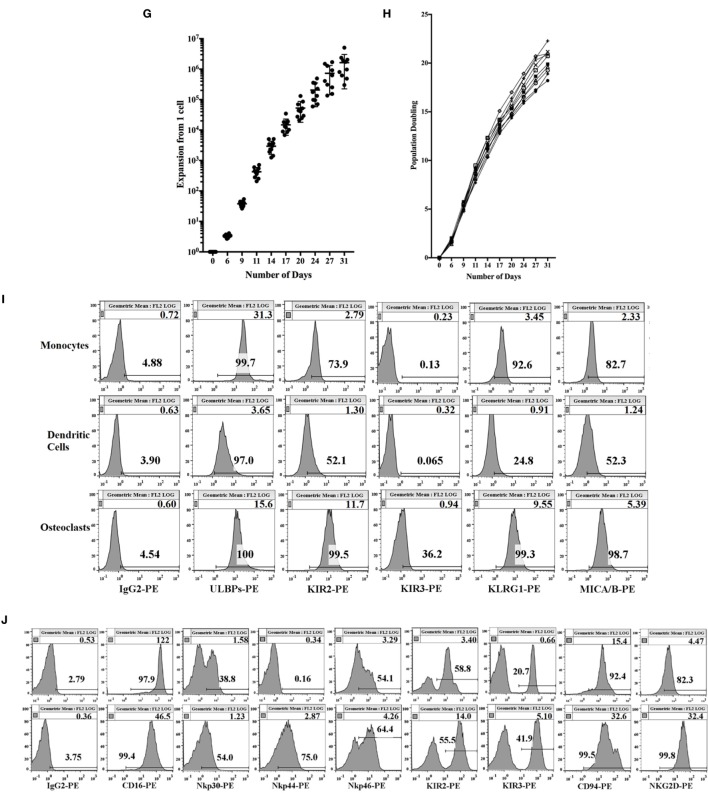
**Expansion of natural killer (NK) cells by osteoclasts (OCs) and T cells by dendritic cells (DCs)**. Monocytes were purified from human PBMCs and were cultured with GM-CSF (150 ng/ml) and interleukin (IL)-4 (50 ng/ml) for 8 days to generate DCs. To generate OCs, monocytes were cultured in alpha-MEM media containing M-CSF (25 ng/ml) and RANKL (25 ng/ml) for 21 days. For expansion, purified NK cells (1 × 10^6^ cells/ml) were treated with the combination of IL-2 (1,000 U/ml) and anti-CD16mAb (3 μg/ml) for 18 h before they were cocultured with autologous monocytes, DCs, or OCs in the presence of sAJ2 at 1:2:4 ratios (monocytes, DCs, or OCs:NK:sAJ2). Surface expression of CD3, CD16, and CD56 was analyzed at days indicated in the figure using flow cytometry, and culture medium was refreshed and supplemented with rh-IL-2 (1,000 U/ml) **(A)**. Cells were cocultured as described in panel **(A)** and the numbers of expanded lymphocytes were assessed using microscopic determination **(B)**. The numbers of NK cells **(C)** and T/NKT **(D)** cells were determined using the percentages of NK and T/NKT cells **(A)** within the total expanding cells in panel **(B)**. Cells were cocultured as described in panel **(A)** and cytotoxicity was determined on the days shown in the figure using the standard 4-h ^51^Cr release assay against the oral squamous carcinoma stem-like cells (OSCSCs). The lytic units 30/10^6^ cells were determined using the inverse number of lymphocytes required to lyse 30% of OSCSCs × 100 **(E)**. Supernatants were harvested from the coculture of NK with OCs as described in panel **(A)** on days 6, 9, 12, and 15, and interferon-γ secretion was determined using single ELISA **(F)**. NK cells were cocultured with autologous OCs and expanded from 10 healthy donors as described in panel **(A)**. Cumulative fold expansion of NK cells was calculated for each donor for 31 days **(G)**, and population doubling was calculated based on the log of the ratio of the final count to the baseline count divided by the log of 2 **(H)**. DCs and OCs were generated as described in panel **(A)** and 1 × 10^4^ cells were used to analyze ULBPs, KIR2, KIR3, killer cell lectin-like receptor G1 (KLRG1), and MICA/B surface expressions using PE-conjugated antibodies and flow cytometric analysis. IgG2 isotype control antibody was used as control **(I)**. Freshly isolated NK cells (upper row) and NK cells cocultured with autologous OCs and expanded as described in panel **(A)** (lower row) were used to analyze CD16, Nkp30, Nkp44, Nkp46, KIR2, KIR3, CD94, and NKG2D surface expression using, PE-conjugated antibodies. IgG2 isotype control antibody was used as controls **(J)**.

**Figure 2:**
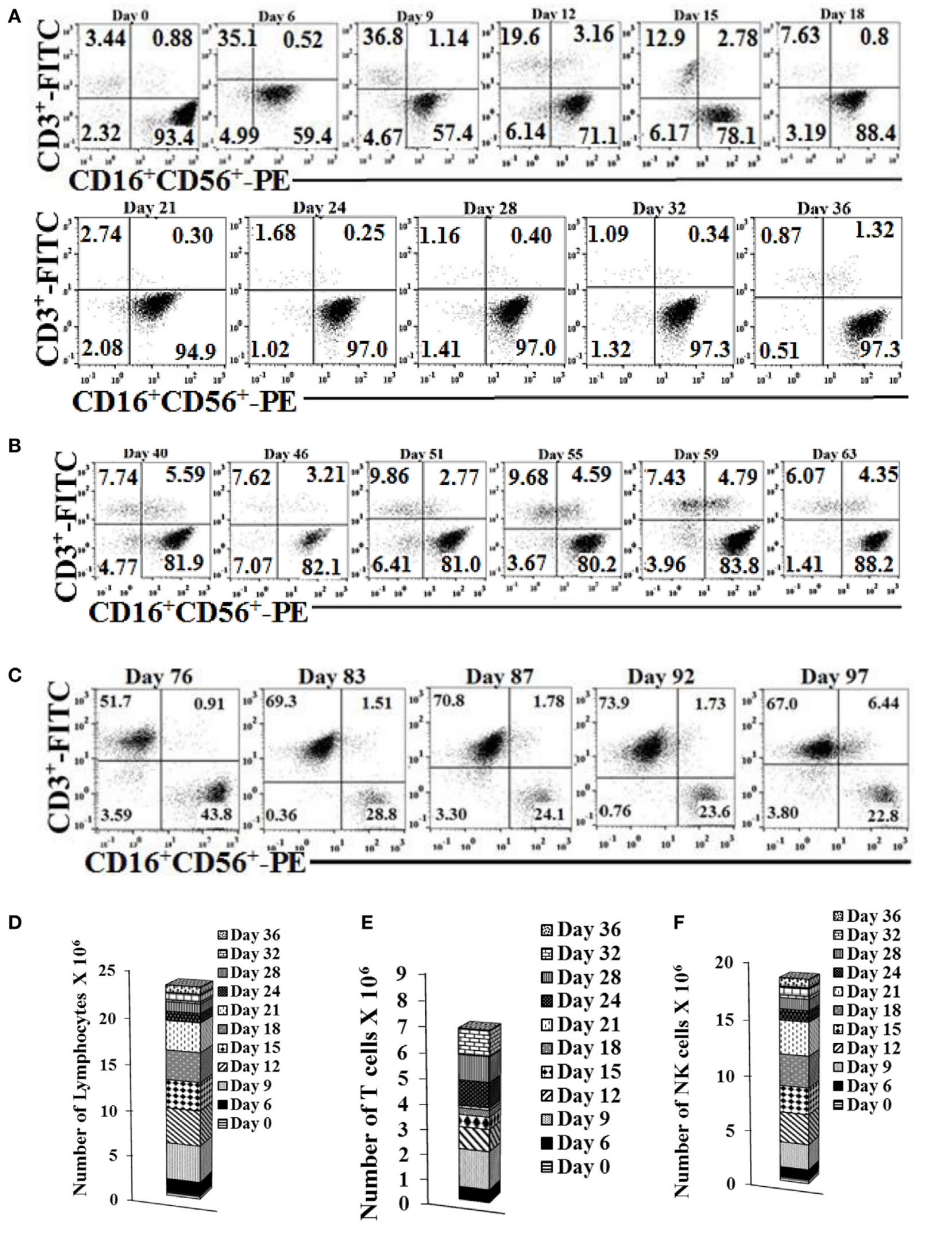

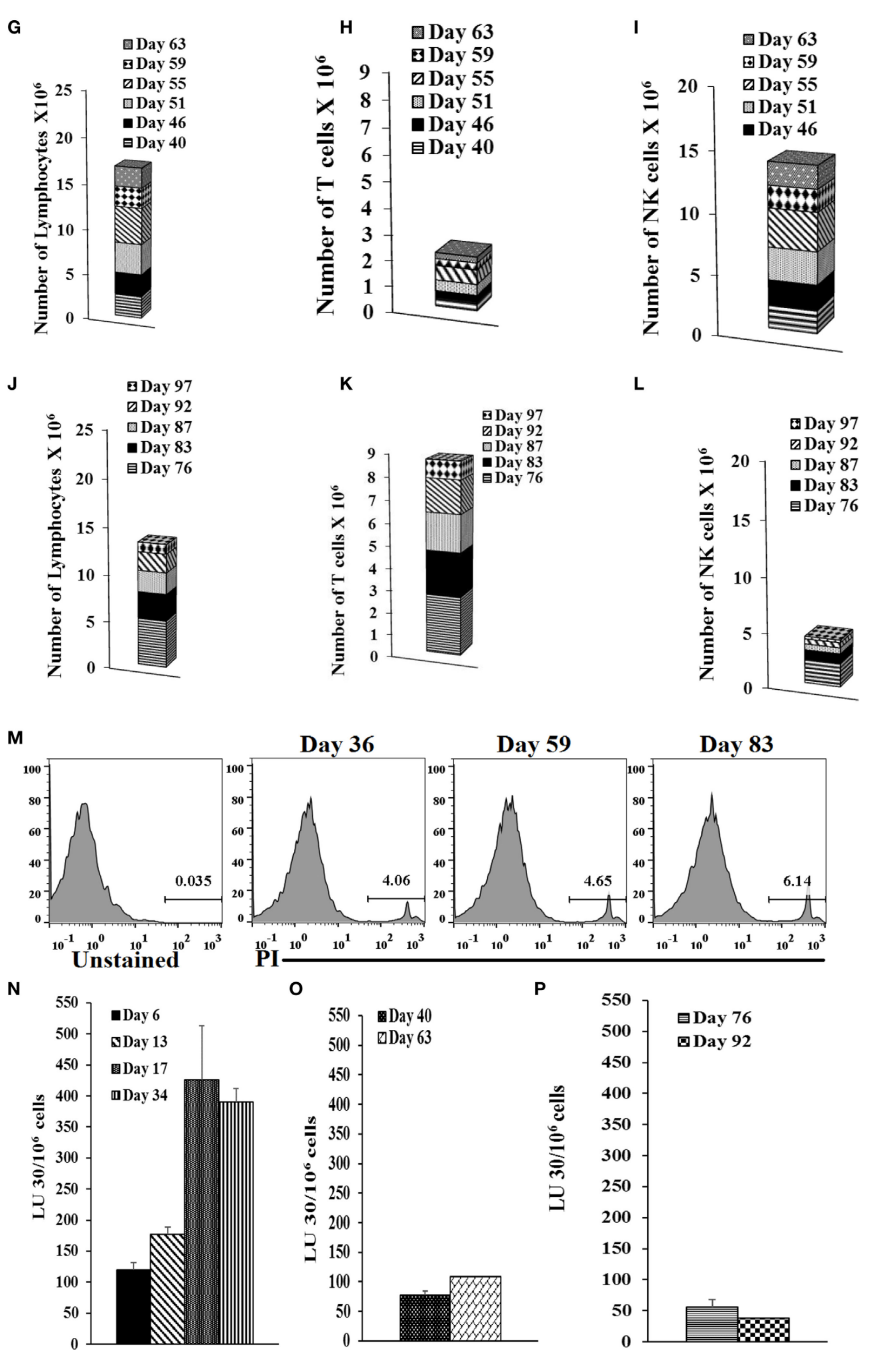

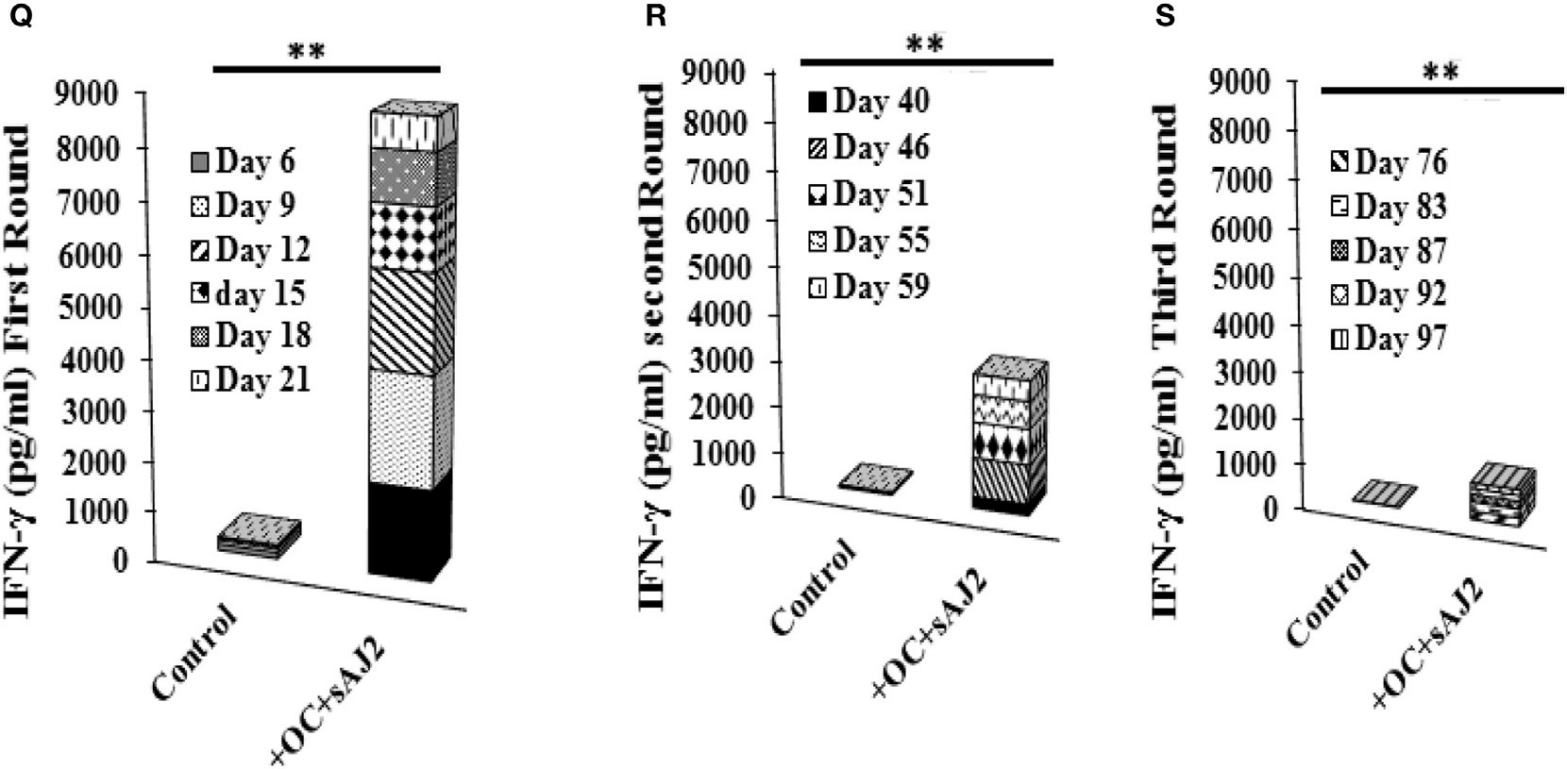
**Reduced proportions of natural killer (NK) cells, NK cell-mediated cytotoxicity, and interferon (IFN)-γ secretion with each successive monthly re-stimulation of NK cell cultures with osteoclasts (OCs) and sAJ2 bacteria**. Freshly purified NK cells were treated and cocultured with monocyte-derived autologous OCs as described in Figure 1A. Surface expression of CD3, CD16 and CD56 was analyzed in 1 × 10^4^ lymphocyte from cocultures at days indicated in the figure using flow cytometric analysis **(A)**. After 36 days, when NK cells ceased to expand, they were re-cultured with fresh autologous OCs as described in Figure 1A. Surface expression of CD3, CD16, and CD56 was analyzed on the days indicated in the figure using antibody staining and flow cytometric analysis **(B)**. On day 63, when cells ceased to expand, they were re-cultured with OCs as described and surface expression of CD3, CD16, and CD56 was analyzed on days shown in the panel **(C)**. The numbers of expanded lymphocytes were assessed using microscopic determination **(D,G,J)** and the numbers of NK cells **(F,I,L)** and T/NKT **(E,H,K)** cells were determined using the percentages of NK and T/NKT cells within the total expanding cells **(D,G,J)**. Cell death was determined in lymphocytes at days 36, 59, and 83 using propodium iodide staining and flow cytometric analysis **(M)**. Freshly purified NK cells were treated and cocultured with autologous OCs as described in Figure 1A. Lymphocytes were then tested for cytotoxicity using the standard 4-h ^51^Cr release assay against the oral squamous carcinoma stem-like cells after 6, 13, 17, and 34 days of the coculture **(N)**, 40 and 63 days of the coculture **(O)**, or 76 and 92 days of the coculture **(P)**. The lytic units 30/10^6^ cells were determined using the method described in Figure 1E. The supernatants were harvested and IFN-γ secretion was determined using single ELISA using supernatants from days 6, 9, 12, 15, 18, and 21, **(Q)**; days 40, 46, 51, 55, and 59 **(R)**, and days 76, 83, 87, 92, and 97 **(S)**.

We compared the activating effect of OCs, monocytes, and DCs on NK cell expansion and function. NK cells were activated with IL-2 and anti-CD16 mAb 18–20 h before their coculture with OCs and/or sAJ2. The combination of OCs and sAJ2 preferentially expanded NK cells while maintaining a low proportion of T cells (Figures S1A,B in Supplementary Material). The rate of expansion and the levels of contaminating T cells in NK cultures were then compared between the cocultures with OCs, DCs, and monocytes treated with sAJ2. NK cells cocultured with OCs preferentially expanded NK cells, and the rate of contaminating T cells remained very low throughout the first 1–2 months of the culture (Figures 1A–D and 2A–F). By contrast, DCs preferentially expanded T cells and the proportion of NK cells remained low. Although monocytes were also able to expand T cells, T cell expansion remained lower than those of NK cells. There was an initial increase in the proportion of T cells in all three cultures; however, in subsequent cultures, the rate of T cell expansion decreased in the cocultures with OCs, whereas T cells continued expanding and were substantially increased in cultures with DCs. A steady state of T cell expansion can also be seen in cocultures with monocytes (Figures 1A,D).

Natural killer cells cocultured with OCs were able to lyse OSCSCs significantly more than NK cells cocultured with monocytes or DCs, and there was a significant increase from day 9 to day 15, correlating with the higher numbers of NK cells in coculture with OCs on day 15 (Figures 1A,C and E). IL-2- and anti-CD16mAb-activated NK cells cultured with OCs secreted significantly higher amounts of interferon (IFN)-γ, compared to NK cells cocultured with monocytes or DCs (Figure 1F and Figure S1C in supplementary material). Upon analysis of NK cell expansion rate and population doubling (defined by the log of the ratio of the final count to the baseline count divided by the log of 2) it was found that OCs expanded NK cells 21,000- to 132,000-fold at day 20 and 300,000- to 5,100,000-fold on day 31, with 17–21 population doublings within 4 weeks (Figures 1G,H).

Freshly isolated monocytes were compared with mature DCs and OCs for expression of key surface receptors. CD54 was upregulated on DCs and OCs, whereas MHC-I was decreased on DCs and OCs, when compared to monocytes (Figure S1D in Supplementary Material). Killer cell immunoglobulin-like receptors (KIRs), killer cell lectin-like receptor G1, and MICA/B were higher on OCs, intermediate on monocytes, and very low on DCs (Figure 1I and Figure S1D in supplementary material). ULBP 1–6 were high on monocytes, intermediate on OCs, and low on DCs (Figure 1I). NK cell receptors including CD94 and NKG2D were higher on OC-expanded NK cells (Figure 1J, lower row) as compared to untreated primary NK cells (Figure 1J, upper row). Expression of CD16 receptor was decreased on expanded NK cells when compared to primary NK cells (Figure 1J).

### Residual Population of T Cells Purified from OC-Expanded NK Cells Did Not Contribute to Lysis by the NK Cells

The majority of T cell contaminants from OC-expanded NK cells were CD8+ T cells (Figure S2A in Supplementary Material). To determine whether contaminating T cells contributed to NK cell mediated killing of OSCSCs or K562s, T cell contaminants from day 9 OC-expanded NK cells were sorted out positively to obtain purified T cells. Purified NK cells or T cells (Figure S2B in Supplementary Material) were then used in ^51^Cr release assay against OSCSCs and K562s. As expected CD3+ T cells isolated from OC-expanded NK cells did not contribute to lysis of OSCSCs (Figure S2C in Supplementary Material) or K562s (Figure S2D in Supplementary Material). In addition, supernatants from NK cells secreted significantly higher levels of IFN-γ compared to T cells (Figure S2E in Supplementary Material).

### Expansion of NK Cells with OCs Remained High in the First and Second Month, and Decreased Substantially in the Third Month

Natural killer cells cultured with OCs expanded for 31–36 days while the percentages of contaminating T cells remained low (Figure 2A). Day 36 expanded NK cells were re-cultured with OCs for a second round of expansion and the NK expansion was continued for 27 days (Figure 2B). Similarly, the percentages of T cell expansion remained very low in the second round of NK cell expansion with OCs (Figure 2B). Day 67 expanded NK cells were re-cultured with OCs for the third round of expansion; however, NK cells were gradually lost due to T cell expansion (Figure 2C). When the total numbers of NK and T cells were determined during expansion, the numbers of NK cells remained high in the first and second month of expansion (Figures 2D–2I) and substantially decreased in the third month (Figures 2J–2L). No or slight levels of cell death could be observed in the expanding NK cells in the three rounds of expansion with OCs (Figure 2M). The ability of NK cells to lyse cancer stem cells and secrete IFN-γ was gradually decreased from the first to second round of expansion, and in the third round, during which greater percentages of T cells expanded, these functions became minimal (Figures 2N–S). Interestingly, even though large numbers of NK cells were still expanding during the second round of stimulation with OCs from days 40–63, the levels of NK cell cytotoxicity and secretion of IFN-γ were substantially lower than those obtained from expanded NK cells in the first round of stimulation on days 0–34 (Figure 2).

### OCs, But Not K562 or OSCSCs, Expand NK Cells and Increase NK Cell Function

Activated NK cells were cultured with OSCSCs, K562, OCs, irradiated K562, or irradiated OCs in the presence of sAJ2 and the levels of NK expansion and their function were determined (Figures S3A–H in Supplementary Material). NK cell expansion and function (cytotoxicity and IFN-γ secretion) induced by either non-irradiated, irradiated K562, or OSCSCs was significantly lower than those induced by non-irradiated or irradiated OCs (Figure S3 in Supplementary Material).

### Decreased Cytotoxicity and Lower IFN-γ Secretion by NK Cells from Patients Coincides with Increased Expansion of T Cells

**Figure 3:**
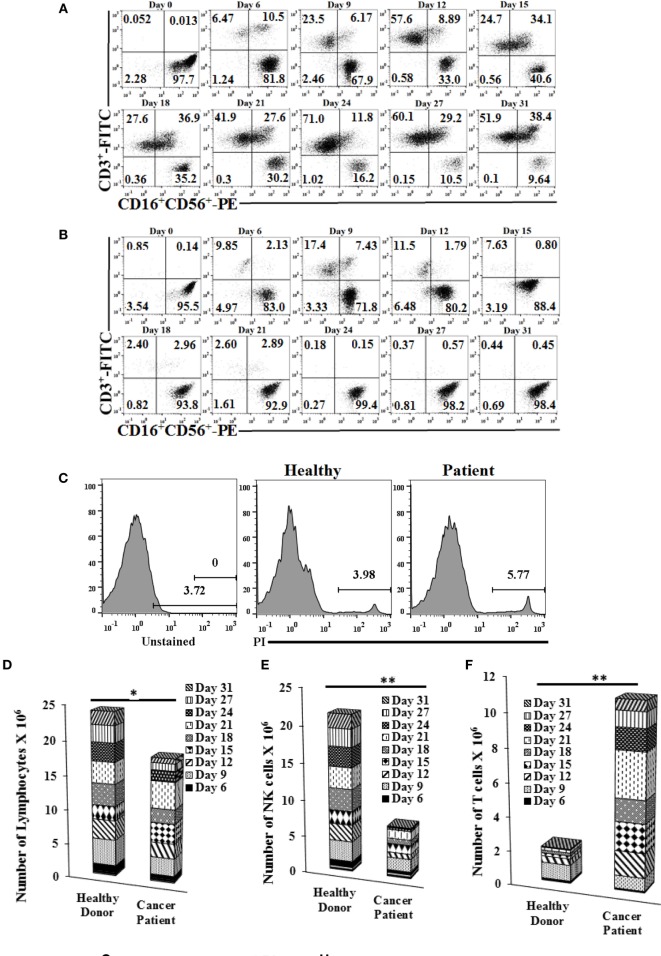

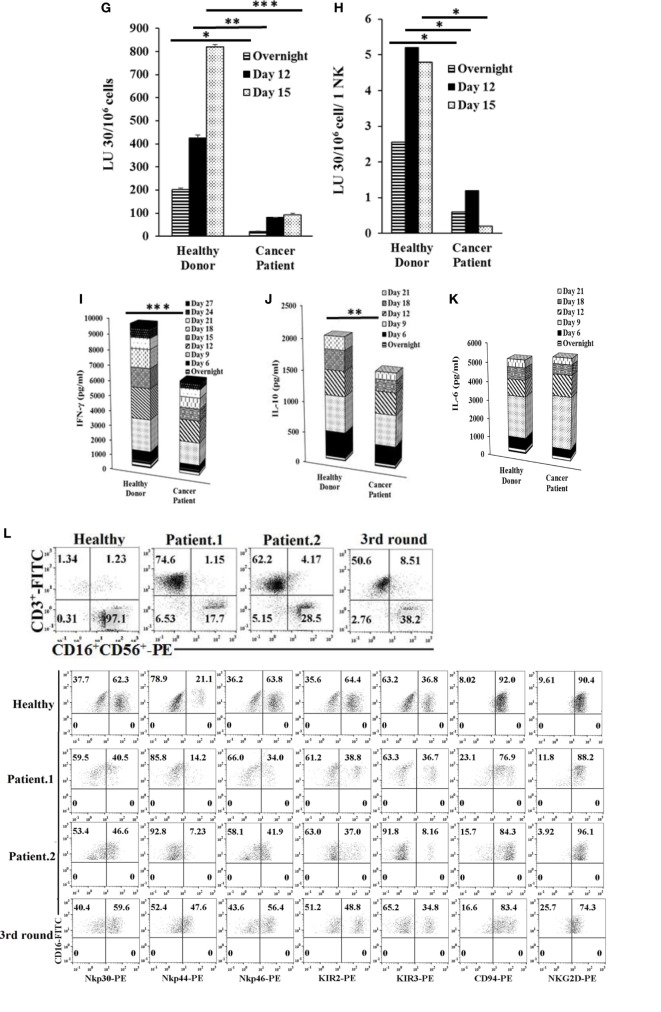

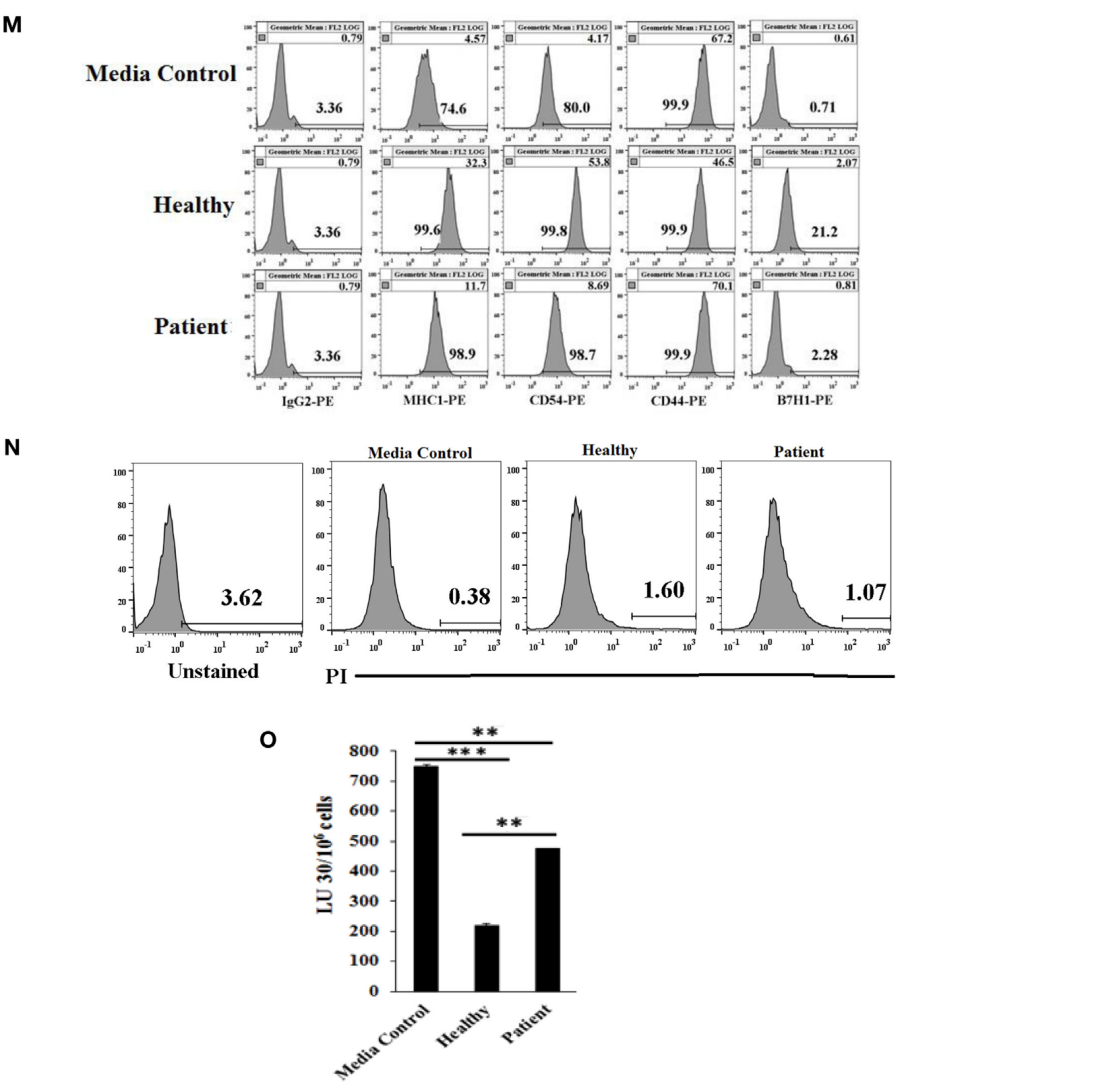
**Purified natural killer (NK) cells cultured with osteoclasts (OCs) from cancer patients expand more T cells than NK cells, mediate much lower cytotoxicity, and cytokine secretion compared to those expanded from healthy donors**. Freshly purified NK cells from healthy donor and cancer patient were treated and cocultured with monocyte-derived OCs as described in Figure 1A. Surface expression of CD3, CD16, and CD56 was analyzed on expanding cells at days 0, 6, 9, 12, 15, 18, 21, 24, 27, and 31 of cancer patient **(A)** and healthy donor **(B)** using antibody staining followed by flow cytometric analysis. Cell death was determined on expanding NK cells from cancer patients and healthy donors at day 19 using propidium iodide (PI) staining and flow cytometric analysis **(C)**. After 6, 9, 12, 15, 18, 21, 24, 27, and 31 days of coculture, expanded lymphocytes were assessed using microscopic determination **(D)**. The numbers of NK cells **(E)** and T/NKT **(F)** cells were determined using the percentages of NK and T/NKT cells within the total expanding cells in panels **(A,B)**. Cytotoxicity of lymphocytes was determined following overnight, 12, and 15 days of coculture using standard 4-h ^51^Cr release assay against oral squamous carcinoma stem-like cells (OSCSCs). The lytic units 30/10^6^ cells were determined using the method described in Figures 1E,G. Lytic units from panel **(G)** were normalized based on per NK cells **(H)**. The supernatants were harvested from the overnight, days 6, 9, 12, 15, 18, 21, 24, and 27 cocultures and IFN-γ **(I)**, interleukin (IL)-10 **(J)**, and IL-6 **(K)** secretion was determined using single ELISAs. Freshly purified NK cells from healthy donors, cancer patient with tonsillar carcinoma (patient.1), and pancreatic cancer (patient.2) were treated and cocultured with OCs as described in Figure 1A. Surface expressions of CD3, CD16, and CD56 were analyzed on lymphocytes from cocultures of day 21 for healthy donor and patient NKs, and day 87 (third stimulation) of healthy donors [**(L)** upper panel], and surface expression of Nkp30, Nkp44, Nkp46, KIR2, KIR3, CD94, and NKG2D was analyzed within CD16 positive cells [**(L)** lower panels]. IgG2 isotype control antibodies were used as controls **(L)**. The supernatants were harvested from the cocultures on day 13, and the equal volumes of supernatants (200 μl) from each donor were used to differentiate OSCSCs for overnight, before the levels of MHC-I, CD54, CD44, and B7H1 surface expressions were determined on OSCSCs. IgG2 isotype control antibodies were used as controls **(M)**. Cell death was determined in untreated and NK cell supernatant-differentiated OSCSCs using propodium iodide staining and flow cytometric analysis **(N)**. Highly purified NK cells were treated with IL-2 (1,000 U/ml) and used to determine cytotoxicity against untreated and NK supernatant-differentiated OSCSCs in 4-h ^51^Cr release assay. The lytic units 30/10^6^ cells were determined using the method described in Figure 1E **(O)**.

When cultured with OCs, purified NK cells from cancer patients in comparison to healthy controls were unable to maintain the expansion of NK cells and indeed, by day 12, greater than half of the expanding cells were T cells. Moreover, by day 31, only 10% of the remaining cells in the culture were NK cells (Figures 3A,B; Figures S4A,B in Supplementary Material). In addition, when total numbers of expanded NK and T cells were determined within 31 days of expansion in cancer patients in comparison to healthy controls, there were less expanding cells from cancer patients when compared to healthy controls, even though no or a slight increase in cell death could be seen in expanding NK cultures in patient cells as compared to healthy controls (Figures 3C,D; Figures S4C–F in Supplementary Material), and the levels of expanding T cells were significantly higher than NK cells (Figures 3E,F; Figures S4D,E in Supplementary Material). By contrast, NK cells isolated from healthy donors maintained the expansion of NK cells and the levels of NK expansion were significantly higher than T cells (Figures 3B,E,F; Figures S4B,D,E in Supplementary Material). No or much lower proliferation of NK cells were observed with patient NK cells when compared to healthy NK cells at different days of culture (Figure 3E; Figure S4D in Supplementary Material).

Patients’ NK cells cultured with OCs lysed OSCSCs significantly less when compared with the healthy NK cells cultured with OCs (Figure 3G; Figure S4G in Supplementary Material). When normalized based on the number of NK cells, cytotoxicity induced per NK cell by patients was less when compared to NK cells from healthy controls (Figure 3H; Figure S4H in Supplementary Material). OC-expanded patient NK cells secreted significantly less IFN-γ when compared to healthy OC-expanded NK cells (Figure 3I; Figure S4I in Supplementary Material). OC-expanded oral cancer patients’ NK cells secreted significantly less IL-10 when compared to healthy NK cells (Figure 3J), whereas those from pancreatic cancer patients secreted higher IL-10 when compared to healthy NK cells (Figure S4J in Supplementary Material). No significant differences could be observed for the levels of IL-6 secretion by healthy or cancer patients NK cells (Figure 3K; Figure S4K in Supplementary Material). Thus, although cultures of NK cells from patients with different tumor types exhibited differences in individual cytokines such as IL-10, they all exhibited lower cytotoxicity and less IFN-γ secretion (Figures 3G–K; Figures S4G–K in supplementary material). The levels of NKG2D surface expression were similar on healthy as compared to patient NK cells expanded by the OCs (Figure 3L). The intensity of CD94 expression is higher on the surface of patient NK cells as compared to healthy control (Figure 3L). KIR2, NKp30, NKp44, and NKp46 expressions were lower on the surface of OC-expanded patient NK cells when compared to healthy NK cells (Figure 3L), whereas KIR3 expression was either the same or lower on the surface of OC-expanded patient NK cells when compared to healthy NK cells (Figure 3L).

### Supernatants from Patient Expanded NK Cells Are Less Able to Differentiate OSCSCs

Oral squamous carcinoma stem-like cells were treated with equal volumes of day 13 supernatants from patient and healthy donors for 18–20 h before the levels of CD44, B7H1, CD54, and MHC-I expressions were analyzed (Figure 3M). There was a 7.1-fold increase in MHC-I expression by healthy NK supernatants but only 2.56-fold increase was induced by patient NK supernatants (Figure 3M). For CD54 expression, a 13-fold increase was observed by healthy NK supernatants compared with a 2.1-fold increase with patient NK supernatants. As for B7H1, a 3.75-fold increase was observed by healthy NK supernatants compared with a 1.5-fold increase with patient NK supernatants. CD44 was decreased by healthy NK supernatants, whereas no decrease was observed by patient NK supernatants (Figure 3M). No significant cell death could be observed after treating OSCSCs with NK supernatants (Figure 3N). As shown in Figure 3O, 74% decrease in NK cell-mediated cytotoxicity was observed when OSCSCs were treated with healthy NK supernatants, whereas only 33% decrease could be observed with patient NK supernatants (Figure 3O).

### Oral Tumors in Humanized Mice Preferentially Expand T Cells Resulting in the Loss of NK Cytotoxicity While Retaining IFN-γ Secretion

**Figure 4:**
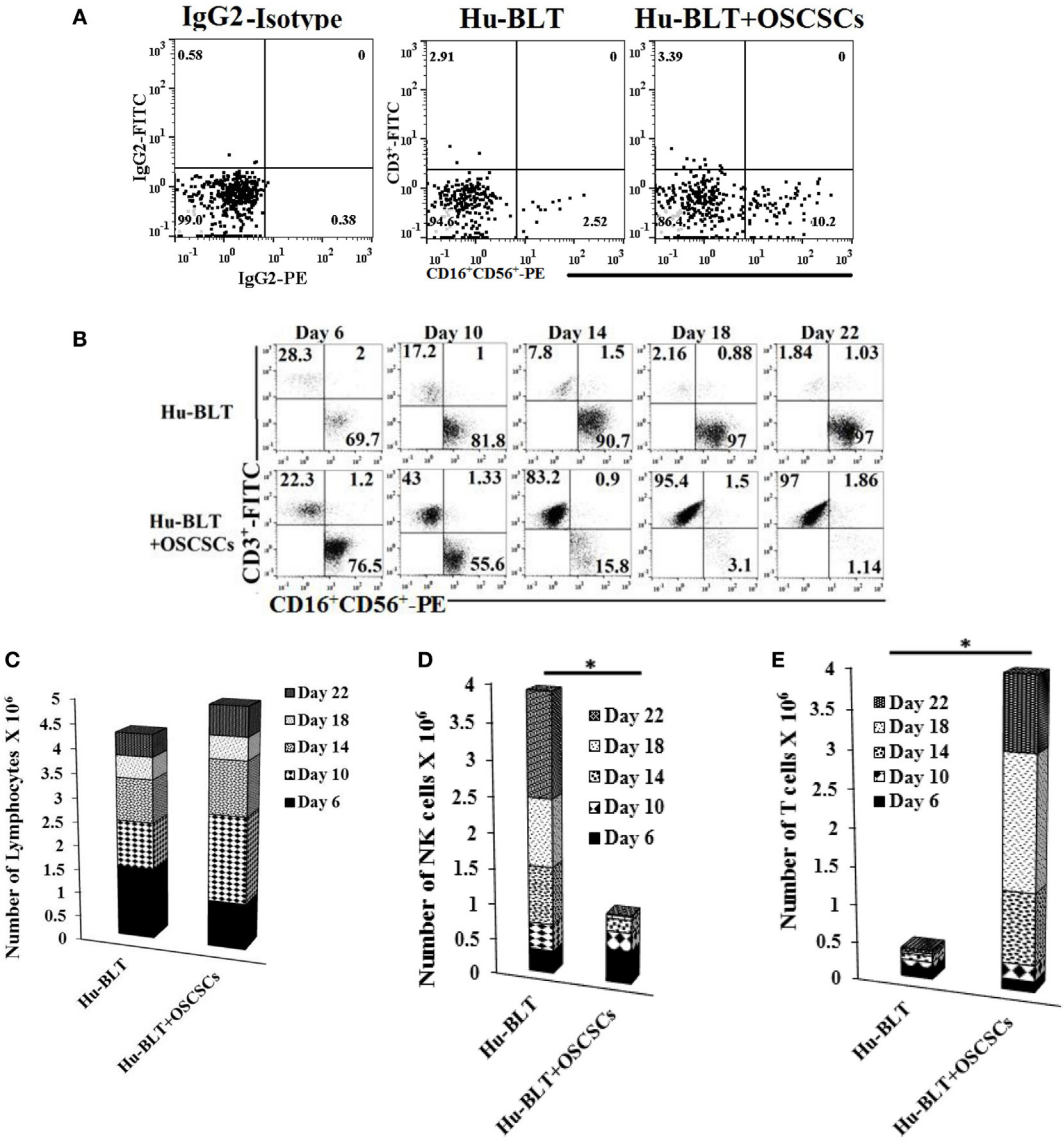

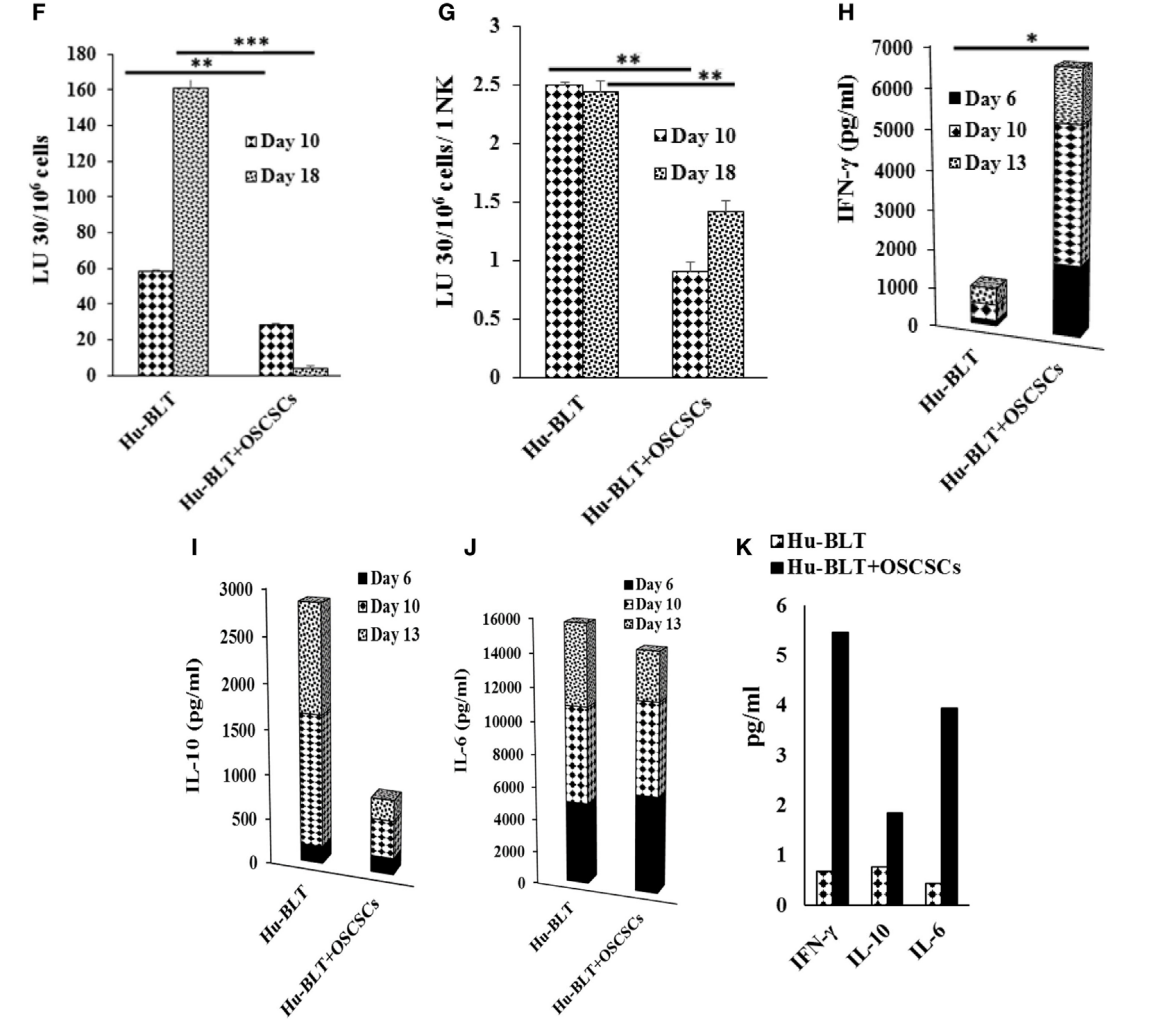
**Spleen natural killer (NK) cells depleted of T cells from tumor-bearing humanized-BLT mice cultured with osteoclasts (OCs) expand small fraction of contaminating T cells faster than NK cells obtained from healthy hu-BLT mice cultured with OCs**. Reconstituted BLT mice were orthotopically injected with 1 × 10^6^ of human oral squamous carcinoma stem-like cells (OSCSCs) into the floor of the mouth. Disease progression and weight loss were monitored for another 4–5 weeks. Mice were sacrificed, spleens were harvested, and single cell suspensions were obtained as described in Section “Materials and Methods.” CD3+ T cells were sorted out using positive selection kit and the flow-through cells were analyzed for surface expression of human CD3/CD16/CD56 after staining with the respective antibodies. Isotype control antibodies were used as controls **(A)**. CD3-negative cells (1 × 10^6^ cells/ml) from hu-BLT mice were treated with the combination of interleukin (IL)-2 (1,000 U/ml) and anti-CD16mAb (3 μg/ml) for 18 h before they were cultured with OCs in the presence of sAJ2 at 1:2:4 ratios (OC:NK:sAJ2). Surface expression of CD3, CD16, and CD56 was analyzed on days 6, 10, 14, 18, and 22 using flow cytometric analysis **(B)**. After 6, 10, 14, 18, and 22 days of coculture, expanded lymphocytes were assessed via microscopic determination **(C)**. The numbers of NK cells **(D)** and T/NKT **(E)** cells were determined using the percentages of NK and T/NKT cells within the total expanding cells. Cytotoxicity of NK cells cocultured for 10 and 18 days was determined using standard 4-h ^51^Cr release assay against OSCSCs and the lytic units 30/10^6^ cells were determined using inverse number of NK cells required to lyse 30% of OSCSCs × 100 **(F)**. Lytic units was normalized and adjusted per NK cell lysis against OSCSCs **(G)**. The supernatants were harvested from the coculture on days 6, 10, and 13, and interferon (IFN)-γ **(H)**, IL-10 **(I)**, and IL-6 **(J)** secretion was determined using single ELISAs. Peripheral blood was collected post-mortem by cardiac puncture from hu-BLT mice and serum samples were harvested and analyzed for IFN-γ, IL-10, and IL-6 secretion using multiplex arrays **(K)**.

Humanized-BLT mice were implanted with oral tumors and mice were sacrificed 4 weeks after tumor implantation. The spleens from hu-BLT mice were harvested, and T cells were sorted out. The flow-through cells containing NK and B cells (Figure S6 in Supplementary Material) were then treated with IL-2 and anti-CD16mAb for 18–20 h before they were cultured with BLT-OCs. Even though tumor-bearing hu-BLT mice contained larger percentages of NK cells (Figure 4A), the expansion resulted in gradual and significant T cell expansion which started on day 6 and continued to day 22, at which point 97% of the cells were T cells and only 1.1% were NK cells. By contrast, flow-through cells from hu-BLT mice with no tumor which contained less NK cells initially, expanded NK cells, and the levels rose from 69.7% NK cells at day 6 to 97% NK cells at day 22 (Figure 4B). The levels of NK cells when cultured with autologous OCs were increased in both animals from the initial day of culture, although mice with no tumor had a 27.66-fold increase from day 0 to day 6, whereas tumor-bearing mice had a 7.5-fold increase (Figure 4B). The total numbers of expanded lymphocytes were slightly higher in tumor-bearing mice as compared to those without tumors (Figure 4C), with the majority of being T cells and not NK cells (Figures 4D,E).

OC-expanded NK cells from tumor-bearing mice were able to lyse OSCSCs significantly less than those of control mice without tumor (Figure 4F). In addition, when cytotoxicity was assessed per NK cell basis, less cytotoxicity was seen with NK cells from tumor-bearing mice as compared to NK cells from control mice without tumor (Figure 4G). OC-expanded NK cells from tumor-bearing mice secreted significantly higher IFN-γ (Figure 4H), lower IL-10 (Figure 4I), and slightly lower IL-6 (Figure 4J) when compared to the control mice without tumor. Sera from peripheral blood of tumor-bearing hu-BLT mice exhibited increased secretion of IFN-γ, IL-10, and IL-6 as compared to the control mice with no tumor (Figure 4K).

### IL-15, in Part, Mediates Expansion of NK Cells by OCs, Whereas IL-12 Is Responsible for Increased IFN-γ Secretion by NK Cells

**Figure 5:**
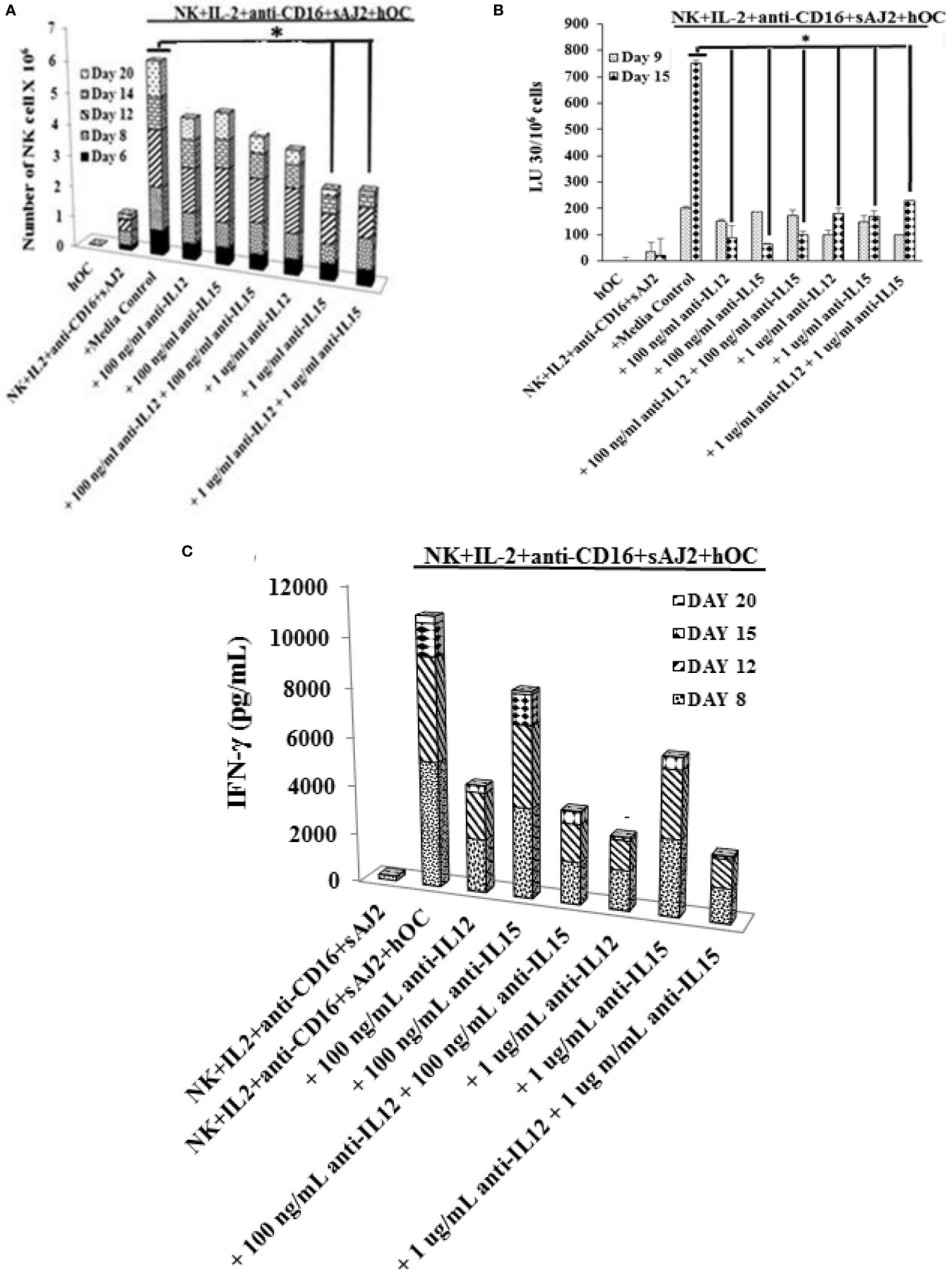
**Blocking interleukin (IL)-12, IL-15, or a combination of both resulted in reduced natural killer (NK) cell expansion, NK cell-mediated cytotoxicity, and cytokine secretion**. Freshly purified NK cells from a healthy donor were treated and cocultured with autologous osteoclasts as described in Figure 1A in the presence and absence of anti-IL-12, -IL-15, or a combination of anti-IL-12 and -IL-15 mAbs at 100 ng/ml and 1 μg/ml, respectively. Cocultures were replenished with IL-2 (1,000 U/ml) every 2 days. NK cells were counted using microscopy on days 6, 8, 12, 14, and 20 **(A)**. On day 9 and day 15, 1 × 10^5^ NK cells from each expanded samples were used in standard 4-h ^51^Cr release against oral squamous carcinoma stem-like cells (OSCSCs). The lytic units 30/10^6^ cells were determined using inverse number of NK cells required to lyse 30% of OSCSCs × 100 **(B)**. The supernatants were harvested from the cocultures on days 8, 12, 15 and 20, and IFN-γ secretion was determined using single ELISA **(C)**.

We determined the levels of cytokines, chemokines, growth factors, and ligands secreted by primary NK cells and day 6 OC-expanded NK cells (Figure S7 in Supplementary Material). The majority of secreted cytokines, chemokines, growth factors, and ligands were higher by OC-expanded NK cells when compared to those secreted by the primary NK cells activated with IL-2 and anti-CD16mAb (Figure S7 in Supplementary Material). A 50–60 fold higher induction of IL-12 and 20- to 26-fold higher IL-15 secretion were seen by OC-expanded NK cells as compared to primary NK cells (Figure S7 in Supplementary Material). Addition of anti-IL-12 and/or anti-IL-15 mAbs significantly reduced cell expansion, with 1 μg/ml of anti-IL-15 having the largest effect (Figure 5A). The treatment with anti-IL-12 and/or anti-IL-15 did not affect the cytotoxic function of NK cells at day 9 (Figure 5B), but cytotoxicity was inhibited significantly at day 15 (Figure 5B). The levels of IFN-γ secretion by OC-expanded NK cells were reduced more by anti-IL-12 when compared to anti-IL-15 treatment (Figure 5C).

### Addition of Anti-CD3 Antibody Controls T Cell Expansion and Increases OC-Expanded NK Cells

**Figure 6:**
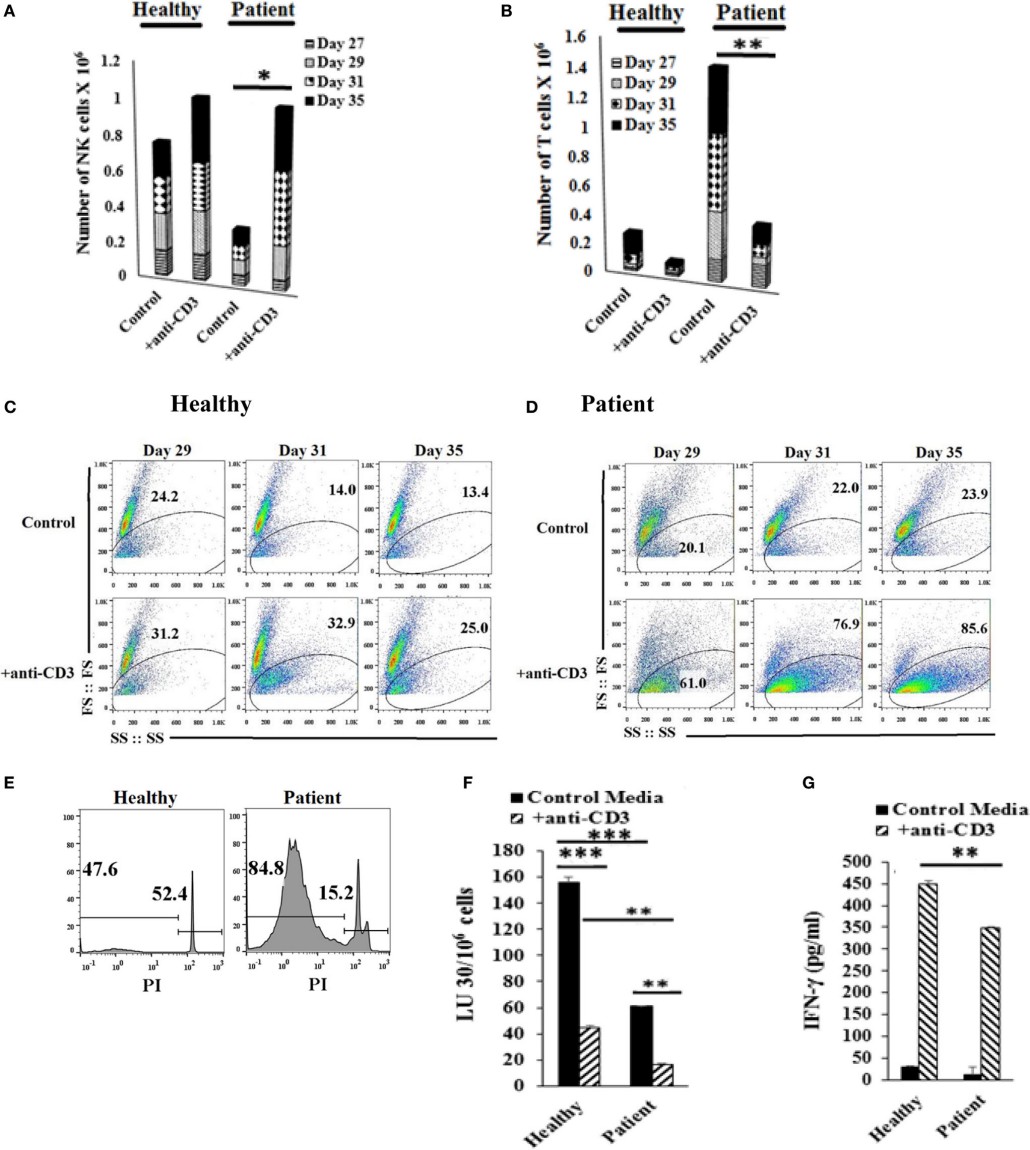
**Addition of anti-CD3 antibody inhibits T cell expansion and increases osteoclast (OC)-expanded natural killer (NK) cells**. Freshly purified NK cells from healthy donors and cancer patients were expanded with OCs for 27 days, the cultures where then treated with 1000 U/mL rh-IL-2 and anti-CD3 (1 μg/ml), before the numbers of NK cells **(A)** and T cells **(B)** were determined by microscopic evaluation on days 27, 29, 31, and 35. Loss of forward and side scatter was determined in healthy donors’ cells **(C)** and patients’ cells **(D)** treated as described in panels **(A,B)**. Healthy donors’ and patients’ cells were stained with propidium iodide (PI) and analyzed for DNA fragmentation **(E)**. Lymphocytes from day 31 culture were used in standard 4-hr ^51^Cr release against oral squamous carcinoma stem-like cells (OSCSCs). The lytic units 30/10^6^ cells were determined using inverse number of NK cells required to lyse 30% of OSCSCs × 100 **(F)**. The supernatants were harvested from the coculture on day 35 and IFN-γ secretion was determined using single ELISA **(G)**.

Lymphocytes were treated with anti-CD3, and NK and T cell expansions were assessed on different days (Figures 6A,B). Loss of forward and side scatter (Figures 6C,D), and elevation in DNA fragmentation as evidenced by an increase in sub G0/G1 peak in cell cycle analysis was obtained in anti-CD3 treated cells indicating loss of T cells (Figure 6E). Accordingly, the levels of NK cells increased in both patient and healthy donors (Figures 6A,B). The population which lost forward and side scatter was CD3+ T cells as determined by CD3 and CD16mAb staining (data not shown). In the absence of NK cells, anti-CD3mAb-treated T cells did not lose forward and side scatter (Figure S8 in Supplementary Material) nor exhibited cell death (data not shown). When cytotoxicity of NK cells was assessed before and after the addition of anti-CD3 mAb, NK cells from both healthy and patient donors had significantly lower cytotoxicity after the addition of anti-CD3mAb, although the cytotoxic function of NK cells were still higher by healthy NK cells when compared to patient NK cells after the addition of anti-CD3mAb (Figure 6F). In contrast, the levels of IFN-γ secretion rose significantly in both healthy and patient NK cells after the addition of anti-CD3mAb indicating the induction of split anergy in NK cells by anti-CD3mAb bound T cells (Figure 6G).

## Discussion

We have recently shown in *in vivo* and *in vitro* studies that OCs are major activators of NK cells (43). More importantly, single monthly stimulation with OCs was able to maintain expansion of super-charged NK cells for over 2 months from healthy donors. In addition, the cytotoxic function of NK cells remained significantly higher during the first month and declined in the second month of expansion. At the present time, it is unclear why NK cell function continues to decline during the second month of stimulation even though large numbers of NK cells continue to expand. It is possible that additional signals are required for the maintenance of NK cell cytotoxicity at the second month and/or more frequent supplementation with OCs is required.

To our knowledge, using OCs as feeder cells is the best strategy for expanding large numbers of NK cells compared to those previously described (Table S1 in Supplementary Material). First, we obtained 21,000- to 132,000-fold expansion on day 20, and 0.3–5.1 million on day 31, with 17–21 population doublings within 4 weeks of expansion, which is a much higher rate than any previously reported method (Table S1 in Supplementary Material). Although cytotoxic function of expanded NK cells across studies is difficult to compare due to different types of targets used, our strategy provides large numbers of NK cells with significant capabilities to target and lyse cancer stem cells/undifferentiated tumors and secrete IFN-γ (Table S1 and Figures S1 and S7 in Supplementary Material, Figure 3, Ref. S13). Moreover, we expand primary NK cells with no or very little chance of transformation, which is known to influence NK cell function since most NK lines (such as YT, NK92, or NK-L) (44) or transfected NK cells tend to either lose all or most or some of their cytotoxic and IFN-γ secretion capabilities, respectively (45, 46). In addition, although anti-CD3 antibody treatment to maintain NK cell expansion would be a good strategy to limit T cell expansion, the cytotoxic activity of such NK cells is lower due to significant split anergy induced in NK cells. This strategy could be great for inducing differentiation of cancer stem cells/undifferentiated tumors due to a larger increase in IFN-γ secretion; however, it may not be sufficient to lyse these tumors. This could be one reason why the use of NK cells in immunotherapy of solid tumors did not result in regression of the tumors in a small numbers of patients tested (46–48).

In contrast to OCs, DCs stimulated preferential expansion of small numbers of contaminating T cells in purified NK cells in healthy donors and the levels of T cells continued to rise for a month thereby decreasing the NK expansion. Accordingly, cytotoxicity was lost against OSCSCs. Interestingly, when the cytotoxicity of NK cells was assessed at an earlier time point, in which similar proportion of NK cells was observed with OC and DC cultures, a rise in NK cytotoxicity could be observed by the NK cells cultured with OCs (data not shown). Similarly, there was an increase in IFN-γ secretion by NK cells cultured with OCs when compared to those cultured with DCs indicating that on a per cell basis, NK cells secreted higher IFN-γ when cultured with OCs as compared to those cultured with DCs (Figure S1C in Supplementary Material). T cells sorted out from OC-expanded NK cells did not contribute to NK cell-mediated cytotoxicity of the NK targets, and the minimal cytotoxicity seen by the T cells was likely due to contaminating NK cells (Figures S2B–D in Supplementary Material). On the other hand, T cells sorted out from OC-expanded NK cells released IFN-γ, but the levels were lower than those released by NK cells (Figure S2E in Supplementary Material). When subsets of T cells: CD8, CD4, and gdT cells were sorted out from PBMCs and used in cytotoxicity assay, only NK cells were able to lyse OSCSCs and the residual cytotoxicity seen by the T cell subsets was due to small numbers of contaminating NK cells (data not shown and Figure S2F in Supplementary Material). OCs in comparison to DCs displayed higher expression of activating NK ligands, whereas they both demonstrated similar levels of differentiation antigen CD54. Interestingly, they both demonstrated lower levels of MHC class I expression when compared to monocytes (Figure S1D in Supplementary Material). In addition, IL-15 secretion appeared to be important for the proliferation of NK cells (Figure 5A) whereas IL-12 was important for the secretion of IFN-γ (Figure 5C). Both cytokines played a key role in cytotoxic function of NK cells when assessed at the later days as compared to earlier time points in expansion (Figure 5B).

In contrast to OCs, all the tumor cell lines tested regardless of whether they were non-irradiated or irradiated did not support the expansion of NK cells long term, and in short term even though secretion of IFN-γ could be observed in cultures with K562 and OSCSCs, this effect was short lived (Figure S3 in Supplementary Material). In agreement, K562 tumors were previously shown to be significantly less effective than EBV lymphoblastoid cell line (R69-LCL) in activating NK cells even in the presence of NK-activating cytokines (49, 50). In addition, OC-induced expansion was also compared to irradiated PBMCs, and it was found to be significantly inferior to that of OCs (data not shown). NK cells expanded by OCs demonstrated much higher levels of activating receptor expression including NKG2D, NKp46, NKp44, NKp30, CD94, and increased inhibitory receptors KIR2 and KIR3 with much lower expression of CD16 receptor when compared to primary NK cells (Figure 1J, lower row). When comparing OC-expanded NK cells from healthy donors, cancer patients had in general much lower receptor expression, and the levels were even less than those seen during third round of stimulated healthy NK cells which had lost significant cytotoxic and cytokine secretion capabilities, and allowed for the expansion of T cells. Patients’ NK cells, similar to NK cells expanded by K562 and OSCSCs were short lived and had much lower cytotoxic and cytokine secretion capabilities. K562 or OSCSCs, unlike OCs, expressed lower levels of NK-activating ligands (Figure S1D in Supplementary Material), and lacked secretion of key cytokines responsible for the expansion of NK cells, since increase in these signals by OCs was able to elevate expansion and function of NK cells (Figure S7A in Supplementary Material). It remains to be determined if such signals by engineered K562s (Table S1 in Supplementary Material) are inferior to those delivered by the OCs since the rate of expansion of NK cells by engineered K562 cells is lower by a magnitude of 100-fold from OC-expanded NK cells (Table S1 and Figures S3A,B in Supplementary Material). In addition, continuous stimulation by the engineered K562s is required to maintain NK expansion whereas only one stimulation with OCs is sufficient to expand super-charged NK cells for over a month (51). Moreover, OC-expanded NK cells, unlike primary NK cells, withstand freezing temperatures quite well and retain their super-charged characteristics and expansion rates with no loss of viability or function (Figure S5 in Supplementary Material).

When OC were used to expand NK cells from cancer patients a very distinct profile was observed. OCs also expanded T cells from purified cancer patients’ NK cells early in the culture with decline in expansion of NK cells at different days. When assessing the function of patients’ NK cells after OC cultures a significant loss of NK cell cytotoxicity, and a decrease in IFN-γ secretion could be observed per NK cells (Figures 3G,I; Figures S4G–I in Supplementary Material). This observation is important since it indicates that faster expansion of contaminating small fraction of T cells in purified NK cultures in cancer patients correlates with loss of cytotoxic function of NK cells. Whether loss of NK cytotoxicity in cancer patients allows for the expansion of T cells, and/or expanding T cells have inhibitory effect on the expansion of patient NK cells requires further investigation. It is also possible that T cells have in general greater survival advantage in comparison to NK cells and are able to proliferate faster than NK cells. These possibilities are presently under investigation in our laboratory. However, it is likely that the loss of NK cells may also provide the fertile ground for the continued growth and eventual metastasis of cancer stem cells.

To test whether OCs obtained from humanized mice implanted with tumors, similar to cancer patients’ OCs expand contaminating small fraction of T cells within purified NK cultures, we implanted OSCSCs in the floor of the mouth. After 5 weeks, mice were euthanized and splenic T cells were depleted before the remaining cells were cultured with autologous and allogeneic OCs and the rate of NK cell expansion were determined. Similar to healthy donors’ NK cells, NK cells from hu-BLT mice without tumor expanded NK cells for a longer period of time, whereas those from tumor-bearing mice expanded the small fraction of contaminating T cells within the NK cultures faster favoring the expansion of T cells. Interestingly, similar to the loss of NK cell cytotoxicity observed in cancer patients we also observed significant loss of NK cell cytotoxicity in hu-BLT mice implanted with tumors which as indicated above may be the underlying mechanism for the expansion of T cells. However, OC-expanded NK + T cells from the tumor-bearing mice secreted higher levels of IFN-γ when compared to those from mice with no tumor, suggesting the potential induction of split anergy in NK cells to drive differentiation of cancer stem cells. This was found to be the case since tumor cells isolated from tumor bearing hu-BLT mice demonstrated higher differentiation antigens including MHC class I and were resistant to NK cell-mediated cytotoxicity (data not shown). Therefore, even though NK cytotoxicity was lost in tumor-bearing BLT mice, higher IFN-γ secretion could partly inhibit the growth and expansion of cancer stem cells by promoting differentiation of these tumors as shown previously (2).

It is hypothesized that the microenvironment in humanized mice is not conducive for the expansion of NK cells due to the lack of cross reactivity between murine and human IL-15, since the addition of human IL-15 promotes an increase in NK cell numbers and function (39). However, if human NK cells are reactive to mouse tissues and, therefore, are rendered hypo-responsive, this may explain why T cells and B cells are significantly expanded in numbers in humanized mice based on our findings. Inactivation or loss of NK function may allow faster and greater expansion of T and B cells at the loss of NK cells as seen with cancer patients’ NK cells. Humanized mice are therefore a good model to study human cancers since in both cases lower function of NK cells may potentially expand T cells at the loss of NK cells.

Rapid expansion of T cells and decreased NK cell numbers in peripheral blood/tissues of cancer patients and in humanized mice could be detrimental for targeting MHC class I low targets including cancer stem cells/undifferentiated tumors by NK cells in order to minimize the tumor load. In addition, NK cells also provide large amounts of IFN-γ to promote optimal differentiation of the cancer stem cells to increase MHC class I expression, thereby paving the road for targeting of NK differentiated tumors by CD8+ T cells. Thus, decline in the numbers and function of NK cells in cancer patients may not only prevent control of cancer stem cell/undifferentiated tumors but it will also decrease expansion of CD8+ T cells leading to an increase in tumor growth. Thus, restoration of NK cell numbers and function in cancer patients will be important to establish effective control of tumor growth.

## Author Contributions

AJ was the principal investigator, obtained the funding, designed the study, and wrote the manuscript along with first author. KK performed all the experiments, analyzed the results, and wrote the manuscript. JC assisted in performing some experiments and edited the manuscript. S-HP performed some of the experiments. PT performed some of the experiments generated the graphs for the manuscript and supplementary material, and edited the manuscript. AK reviewed and edited the manuscript. NO generated graphs and edited the manuscript. CF reviewed and edited the manuscript. IN reviewed and edited the manuscript.

## Conflict of Interest Statement

The authors declare that the research was conducted in the absence of any commercial or financial relationships that could be construed as a potential conflict of interest.

## Abbreviations

CSCs, cancer stem-like cells; DCs, dendritic cells; FBS, fetal bovine serum; HLA, human leukocyte antigen; Hr, human recombinant; IFN, interferon; IL, interleukin; KIR, killer cell immunoglobulin-like receptor; KLRG1, killer cell lectin-like receptor G1; MHC, major histocompatibility complex; NK, natural killer; OCs, osteoclasts; OSCSCs, oral squamous carcinoma stem-like cells.

## References

[1] Jewett A Man YG Cacalano N Kos J Tseng HC. Natural killer cells as effectors of selection and differentiation of stem cells: role in resolution of inflammation. J Immunotoxicol (2014) 11(4):297–307. 10.3109/1547691X.2013.877104 24575813

[2] Tseng HC Bui V Man YG Cacalano N Jewett A. Induction of split anergy conditions natural killer cells to promote differentiation of stem cells through cell-cell contact and secreted factors. Front Immunol (2014) 5:269. 10.3389/fimmu.2014.00269 24995006 PMC4062968

[3] Burke S Lakshmikanth T Colucci F Carbone E. New views on natural killer cell-based immunotherapy for melanoma treatment. Trends Immunol (2010) 31(9):339–45. 10.1016/j.it.2010.06.003 20655806

[4] Larsen SK Gao Y Basse PH NK cells in the tumor microenvironment. Crit Rev Oncog (2014) 19(1–2):91–105. 10.1615/CritRevOncog.2014011142 24941376 PMC4062922

[5] Imai K Matsuyama S Miyake S Suga K Nakachi K. Natural cytotoxic activity of peripheral-blood lymphocytes and cancer incidence: an 11-year follow-up study of a general population. Lancet (2000) 356(9244):1795–9. 10.1016/S0140-6736(00)03231-1 11117911

[6] Bruno A Ferlazzo G Albini A Noonan DM. A think tank of TINK/TANKs: tumor-infiltrating/tumor-associated natural killer cells in tumor progression and angiogenesis. J Natl Cancer Inst (2014) 106(8):dju200. 10.1093/jnci/dju200 25178695 PMC4344546

[7] Gross E Sunwoo JB Bui JD Cancer immunosurveillance and immunoediting by natural killer cells. Cancer J (2013) 19(6):483–9. 10.1097/PPO.0000000000000005 24270347

[8] Mirjacic Martinovic KM Babovic N Dzodic RR Jurisic VB Tanic NT Konjevic GM Decreased expression of NKG2D, NKp46, DNAM-1 receptors, and intracellular perforin and STAT-1 effector molecules in NK cells and their dim and bright subsets in metastatic melanoma patients Melanoma Res (2014) 24(4):295–304. 10.1097/CMR.0000000000000072 24769842

[9] Gubbels JA Felder M Horibata S Belisle JA Kapur A Holden H MUC16 provides immune protection by inhibiting synapse formation between NK and ovarian tumor cells. Mol Cancer (2010) 9:11. 10.1186/1476-4598-9-11 20089172 PMC2818693

[10] Balsamo M Scordamaglia F Pietra G Manzini C Cantoni C Boitano M Melanoma-associated fibroblasts modulate NK cell phenotype and antitumor cytotoxicity. Proc Natl Acad Sci U S A (2009) 106(49):20847–52. 10.1073/pnas.0906481106 19934056 PMC2791633

[11] Castriconi R Cantoni C Della Chiesa M Vitale M Marcenaro E Conte R Transforming growth factor beta 1 inhibits expression of NKp30 and NKG2D receptors: consequences for the NK-mediated killing of dendritic cells. Proc Natl Acad Sci U S A (2003) 100(7):4120–5. 10.1073/pnas.0730640100 12646700 PMC153058

[12] Pietra G Manzini C Rivara S Vitale M Cantoni C Petretto A Melanoma cells inhibit natural killer cell function by modulating the expression of activating receptors and cytolytic activity. Cancer Res (2012) 72(6):1407–15. 10.1158/0008-5472.CAN-11-2544 22258454

[13] Krockenberger M Dombrowski Y Weidler C Ossadnik M Honig A Hausler S Macrophage migration inhibitory factor contributes to the immune escape of ovarian cancer by down-regulating NKG2D. J Immunol (2008) 180(11):7338–48. 10.4049/jimmunol.180.11.7338 18490733 PMC3607742

[14] Vitale M Cantoni C Pietra G Mingari MC Moretta L. Effect of tumor cells and tumor microenvironment on NK-cell function. Eur J Immunol (2014) 44(6):1582–92. 10.1002/eji.201344272 24777896

[15] Gallois A Silva I Osman I Bhardwaj N Reversal of natural killer cell exhaustion by TIM-3 blockade. Oncoimmunology (2014) 3(12):e946365 10.4161/21624011.2014.946365 25964857 PMC4353130

[16] Hersey P Edwards A Honeyman M McCarthy WH. Low natural-killer-cell activity in familial melanoma patients and their relatives. Br J Cancer (1979) 40(1):113–22. 10.1038/bjc.1979.147 314301 PMC2009943

[17] Perussia B Ramoni C Anegon I Cuturi MC Faust J Trinchieri G. Preferential proliferation of natural killer cells among peripheral blood mononuclear cells cocultured with B lymphoblastoid cell lines. Nat Immun Cell Growth Regul (1987) 6(4):171–88. 2960890

[18] Rabinowich H Sedlmayr P Herberman RB Whiteside TL. Increased proliferation, lytic activity, and purity of human natural killer cells cocultured with mitogen-activated feeder cells. Cell Immunol (1991) 135(2):454–70. 10.1016/0008-8749(91)90290-R 1709827

[19] Igarashi T Wynberg J Srinivasan R Becknell B McCoy JP Jr Takahashi Y Enhanced cytotoxicity of allogeneic NK cells with killer immunoglobulin-like receptor ligand incompatibility against melanoma and renal cell carcinoma cells. Blood (2004) 104(1):170–7. 10.1182/blood-2003-12-4438 15016654

[20] Srivastava S Lundqvist A Childs RW. Natural killer cell immunotherapy for cancer: a new hope. Cytotherapy (2008) 10(8):775–83. 10.1080/14653240802648181 19089686 PMC7213758

[21] Gras Navarro A Björklund A Chekenya M. Therapeutic potential and challenges of natural killer cells in treatment of solid tumors. Front Immunol (2015) 6:202. 10.3389/fimmu.2015.00202 25972872 PMC4413815

[22] Alici E Sutlu T Bjorkstrand B Gilljam M Stellan B Nahi H Autologous antitumor activity by NK cells expanded from myeloma patients using GMP-compliant components. Blood (2008) 111(6):3155–62. 10.1182/blood-2007-09-110312 18192509

[23] Fujisaki H Kakuda H Shimasaki N Imai C Ma J Lockey T Expansion of highly cytotoxic human natural killer cells for cancer cell therapy. Cancer Res (2009) 69(9):4010–7. 10.1158/0008-5472.CAN-08-3712 19383914 PMC2716664

[24] Berg M Lundqvist A McCoy P Jr Samsel L Fan Y Tawab A Clinical-grade ex vivo-expanded human natural killer cells up-regulate activating receptors and death receptor ligands and have enhanced cytolytic activity against tumor cells. Cytotherapy (2009) 11(3):341–55. 10.1080/14653240902807034 19308771 PMC2736058

[25] Garg TK Szmania SM Khan JA Hoering A Malbrough PA Moreno-Bost A Highly activated and expanded natural killer cells for multiple myeloma immunotherapy. Haematologica (2012) 97(9):1348–56. 10.3324/haematol.2011.056747 22419581 PMC3436235

[26] Imai C Iwamoto S Campana D. Genetic modification of primary natural killer cells overcomes inhibitory signals and induces specific killing of leukemic cells. Blood (2005) 106(1):376–83. 10.1182/blood-2004-12-4797 15755898 PMC1895123

[27] Lapteva N Durett AG Sun J Rollins LA Huye LL Fang J Large-scale ex vivo expansion and characterization of natural killer cells for clinical applications. Cytotherapy (2012) 14(9):1131–43. 10.3109/14653249.2012.700767 22900959 PMC4787300

[28] Koepsell SA Miller JS McKenna DH Jr Natural killer cells: a review of manufacturing and clinical utility. Transfusion (2013) 53(2):404–10. 10.1111/j.1537-2995.2012.03724.x 22670662

[29] Miller JS Soignier Y Panoskaltsis-Mortari A McNearney SA Yun GH Fautsch SK Successful adoptive transfer and in vivo expansion of human haploidentical NK cells in patients with cancer. Blood (2005) 105(8):3051–7. 10.1182/blood-2004-07-2974 15632206

[30] Iliopoulou EG Kountourakis P Karamouzis MV Doufexis D Ardavanis A Baxevanis CN A phase I trial of adoptive transfer of allogeneic natural killer cells in patients with advanced non-small cell lung cancer. Cancer Immunol Immunother (2010) 59(12):1781–9. 10.1007/s00262-010-0904-3 20703455 PMC11030924

[31] Sotiropoulou PA Perez SA Gritzapis AD Baxevanis CN Papamichail M. Interactions between human mesenchymal stem cells and natural killer cells. Stem Cells (2006) 24(1):74–85. 10.1634/stemcells.2004-0359 16099998

[32] Geller MA Cooley S Judson PL Ghebre R Carson LF Argenta PA A phase II study of allogeneic natural killer cell therapy to treat patients with recurrent ovarian and breast cancer. Cytotherapy (2011) 13(1):98–107. 10.3109/14653249.2010.515582 20849361 PMC3760671

[33] Tseng HC Arasteh A Paranjpe A Teruel A Yang W Behel A Increased lysis of stem cells but not their differentiated cells by natural killer cells; de-differentiation or reprogramming activates NK cells. PLoS One (2010) 5(7):e11590. 10.1371/journal.pone.0011590 20661281 PMC2905395

[34] Tseng HC Inagaki A Bui VT Cacalano N Kasahara N Man YG Differential targeting of stem cells and differentiated glioblastomas by NK cells. J Cancer (2015) 6(9):866–76. 10.7150/jca.11527 26284138 PMC4532984

[35] Bui VT Tseng H-C Maung PO Kozlowska A Mann K Topchyan P Augmented IFN-γ and TNF-α induced by probiotic bacteria in NK cells mediate differentiation of stem-like tumors leading to inhibition of tumor growth and reduction in inflammatory cytokine release; regulation by IL-10. Front Immunol (2015) 6:576 10.3389/fimmu.2015.00576 26697005 PMC4667036

[36] Jewett A Bonavida B. Target-induced inactivation and cell death by apoptosis in a subset of human NK cells. J Immunol (1996) 156(3):907–15. 8558016

[37] Shimizu S Hong P Arumugam B Pokomo L Boyer J Koizumi N A highly efficient short hairpin RNA potently down-regulates CCR5 expression in systemic lymphoid organs in the hu-BLT mouse model. Blood (2010) 115(8):1534–44. 10.1182/blood-2009-04-215855 20018916 PMC2830759

[38] Vatakis DN Koya RC Nixon CC Wei L Kim SG Avancena P Antitumor activity from antigen-specific CD8 T cells generated in vivo from genetically engineered human hematopoietic stem cells. Proc Natl Acad Sci U S A (2011) 108(51):E1408–16. 10.1073/pnas.1115050108 22123951 PMC3251070

[39] Kozlowska AK Kaur K Topchyan P Jewett A. Adoptive transfer of osteoclast-expanded natural killer cells for immunotherapy targeting cancer stem-like cells in humanized mice. Cancer Immunol Immunother (2016) 65(7):835–45. 10.1007/s00262-016-1822-9 27034236 PMC4958457

[40] Jewett A Cavalcanti M Bonavida B. Pivotal role of endogenous TNF-alpha in the induction of functional inactivation and apoptosis in NK cells. J Immunol (1997) 159(10):4815–22. 9366406

[41] Jewett A Bonavida B. Interferon-alpha activates cytotoxic function but inhibits interleukin-2-mediated proliferation and tumor necrosis factor-alpha secretion by immature human natural killer cells. J Clin Immunol (1995) 15(1):35–44. 10.1007/BF01489488 7759599

[42] Jewett A Wang MY Teruel A Poupak Z Bostanian Z Park NH. Cytokine dependent inverse regulation of CD54 (ICAM1) and major histocompatibility complex class I antigens by nuclear factor kappaB in HEp2 tumor cell line: effect on the function of natural killer cells. Hum Immunol (2003) 64(5):505–20. 10.1016/S0198-8859(03)00039-9 12691701

[43] Tseng HC Kanayama K Kaur K Park SH Park S Kozlowska A Bisphosphonate-induced differential modulation of immune cell function in gingiva and bone marrow in vivo: role in osteoclast-mediated NK cell activation. Oncotarget (2015) 6(24):20002–25. 10.18632/oncotarget.4755 26343372 PMC4652983

[44] Tam YK Martinson JA Doligosa K Klingemann HG. Ex vivo expansion of the highly cytotoxic human natural killer-92 cell-line under current good manufacturing practice conditions for clinical adoptive cellular immunotherapy. Cytotherapy (2003) 5(3):259–72. 10.1080/14653240310001523 12850795

[45] Magister S Tseng HC Bui VT Kos J Jewett A. Regulation of split anergy in natural killer cells by inhibition of cathepsins C and H and cystatin F. Oncotarget (2015) 6(26):22310–27. 10.18632/oncotarget.4208 26247631 PMC4673165

[46] Perisic Nanut M Sabotic J Jewett A Kos J. Cysteine cathepsins as regulators of the cytotoxicity of NK and T cells. Front Immunol (2014) 5:616. 10.3389/fimmu.2014.00616 25520721 PMC4251435

[47] Cantoni C Huergo-Zapico L Parodi M Pedrazzi M Mingari MC Moretta A NK cells, tumor cell transition, and tumor progression in solid malignancies: new hints for NK-based immunotherapy? J Immunol Res (2016) 2016:4684268. 10.1155/2016/4684268 27294158 PMC4880686

[48] Tallerico R Garofalo C Carbone E. A new biological feature of natural killer cells: the recognition of solid tumor-derived cancer stem cells. Front Immunol (2016) 7:179. 10.3389/fimmu.2016.00179 27242786 PMC4861715

[49] Sanchez-Martinez D Azaceta G Muntasell A Aguilo N Nunez D Galvez EM Human NK cells activated by EBV+ lymphoblastoid cells overcome anti-apoptotic mechanisms of drug resistance in haematological cancer cells. Oncoimmunology (2015) 4(3):e991613. 10.4161/2162402X.2014.991613 25949911 PMC4404803

[50] Sanchez-Martinez D Lanuza PM Gomez N Muntasell A Cisneros E Moraru M Activated allogeneic NK cells preferentially kill poor prognosis B-cell chronic lymphocytic leukemia cells. Front Immunol (2016) 7:454. 10.3389/fimmu.2016.00454 27833611 PMC5081347

[51] Kamiya T Chang YH Campana D Expanded and activated natural killer cells for immunotherapy of hepatocellular carcinoma. Cancer Immunol Res (2016) 4(7):574–81. 10.1158/2326-6066.CIR-15-0229 27197065

