# Supplementary material for: Novel Strategy to Expand Super-Charged NK Cells with Significant Potential to Lyse and Differentiate Cancer Stem Cells: Differences in NK Expansion and Function between Healthy and Cancer Patients
Source: Front Immunol. 2017 Apr 5;8:297. doi: 10.3389/fimmu.2017.00297 (PMC5380683; doi:10.3389/fimmu.2017.00297)
Supplement: Supplementary file 1 [file data_sheet_1.pdf]

Supplementary Information

Table. S1:

**NK Source**	**Method of selection**	**Feeder cells**	**Stimulation**	**Expansion**	**References**
PBMC	Negative selection for NK	Autologous and allogeneic osteoclasts	IL-2, anti-CD16 mAb, sAJ2	21,000-132,000 fold expansion at 20 days, 0.3-5.1 million at 31 days;<br>17-21 population doublings with an average of 19 population doublings within 4 weeks	Current paper
PBMC	Whole PBMCs	None	IL-2, anti-CD16Ab-immobilized flask, OK432	637–5712 fold at 21 days	[6]
PBMC (negative selection)	Negative selection for NK	Irradiated autologous PBMC's (pretreated for 3-5 days with IL2, IL-15, and concanavalin A)	IL-2 and IL-15, (phytohemagglutinin (PHA) and ionomycin first 24 hours)	100 fold at 16 days	[7]
PBMC	CD3 depletion of PBMCs	Autologous irradiated PBMCs stimulated with OKT3	IL-2	79-322 fold at 21-26 days	[8]
PBMC	Whole PBMCs	Wilms tumor cell line (HFWT)	IL-2	58-401-fold at 10-21 days	[9]

PBMC	Whole PBMCs	K562-mb15-41BBL	IL-2	165 (4-567) fold at 14 days	[10]
PBMC	Whole PBMCs	K562-mb15-41BBL	IL-2	21.6 (5.1-86.6) fold at 7 days	[11]
PBMC	Whole PBMCs	K562-GM (transmembrane expression of IL-15, 4-1BBL, and IL-18)	IL-2	500 fold at 21 days	[12]
PBMC	CD3 depletion followed by CD56+ selection	Irradiated EBV-TM-LCL	IL-2	490 (+/- 260) at 21 days	[13]
Cord Blood (CB)	CD34+ positive selection	None	SCF, TPO, IL-7, Fit3L, IL-15, IL-2, G-CSF, GM-CSF, IL-6, LIF, MIP-1α	>15,000 fold at 5 weeks	[14]
Cord Blood (CB)	CD34+ positive selection	None	SCF, Fit3L, TPO, IL-7, GM-CSF, G-CSF, IL-6, IL-15, LMWH, and IL-2	>2,000 fold at 6 weeks (using GMP)	[15]
NK-92	N/A (cell line)	None	IL-2	218- 250 fold at 15-17 days	[16]
PBMC (genetically modified with TERT for immortalization)	Whole PBMCs used initially, residual T-cells removed after 7 days using anti-CD3 Dynabeads	K562-mb15-41BBL (continued stimulation)	IL-2	TERT transformed (130-227 population doublings over 1000 days)<br>Non-transformed (11-20 population doublings at 8-15 weeks)	[17]

PBMCs (after 7 days of culture, retrovirally transduced with NKG2D-DAP10-CD3ζ)	Whole PBMCs used initially, residual T-cells removed after 7 days using anti-CD3 Dynabeads	K562-mb15-41BBL	IL-2	N/A	[18]

1. Bui VT, Tseng HC, Kozlowska A, Maung PO, Kaur K, Topchyan P, Jewett A: **Augmented IFN-gamma and TNF-alpha Induced by Probiotic Bacteria in NK Cells Mediate Differentiation of Stem-Like Tumors Leading to Inhibition of Tumor Growth and Reduction in Inflammatory Cytokine Release; Regulation by IL-10**. *Front Immunol* 2015, **6**:576.
2. Jewett A, Bonavida B: **Target-induced inactivation and cell death by apoptosis in a subset of human NK cells**. *Journal of immunology (Baltimore, Md : 1950)* 1996, **156**(3):907-915.
3. Jewett A, Cavalcanti M, Bonavida B: **Pivotal role of endogenous TNF-alpha in the induction of functional inactivation and apoptosis in NK cells**. *Journal of immunology (Baltimore, Md : 1950)* 1997, **159**(10):4815-4822.
4. Jewett A, Bonavida B: **Interferon-alpha activates cytotoxic function but inhibits interleukin-2-mediated proliferation and tumor necrosis factor-alpha secretion by immature human natural killer cells**. *Journal of clinical immunology* 1995, **15**(1):35-44.
5. Jewett A, Wang MY, Teruel A, Poupak Z, Bostanian Z, Park NH: **Cytokine dependent inverse regulation of CD54 (ICAM1) and major histocompatibility complex class I antigens by nuclear factor kappaB in HEp2 tumor cell line: effect on the function of natural killer cells**. *Human immunology* 2003, **64**(5):505-520.
6. Deng X, Terunuma H, Nieda M, Xiao W, Nicol A: **Synergistic cytotoxicity of ex vivo expanded natural killer cells in combination with monoclonal antibody drugs against cancer cells**. *International immunopharmacology* 2012, **14**(4):593-605.
7. Luhm J, Brand JM, Koritke P, Hoppner M, Kirchner H, Frohn C: **Large-scale generation of natural killer lymphocytes for clinical application**. *Journal of hematotherapy & stem cell research* 2002, **11**(4):651-657.
8. Parkhurst MR, Riley JP, Dudley ME, Rosenberg SA: **Adoptive transfer of autologous natural killer cells leads to high levels of circulating natural killer cells but does not mediate tumor regression**. *Clinical cancer research : an official journal of the American Association for Cancer Research* 2011, **17**(19):6287-6297.

9. Harada H, Saijo K, Watanabe S, Tsuboi K, Nose T, Ishiwata I, Ohno T: **Selective expansion of human natural killer cells from peripheral blood mononuclear cells by the cell line, HFWT**. *Japanese journal of cancer research : Gann* 2002, **93**(3):313-319.
10. Voskens CJ, Watanabe R, Rollins S, Campana D, Hasumi K, Mann DL: **Ex-vivo expanded human NK cells express activating receptors that mediate cytotoxicity of allogeneic and autologous cancer cell lines by direct recognition and antibody directed cellular cytotoxicity**. *Journal of experimental & clinical cancer research : CR* 2010, **29**:134.
11. Fujisaki H, Kakuda H, Shimasaki N, Imai C, Ma J, Lockey T, Eldridge P, Leung WH, Campana D: **Expansion of highly cytotoxic human natural killer cells for cancer cell therapy**. *Cancer research* 2009, **69**(9):4010-4017.
12. Yang H, Tang R, Li J, Liu Y, Ye L, Shao D, Jin M, Huang Q, Shi J: **A New Ex Vivo Method for Effective Expansion and Activation of Human Natural Killer Cells for Anti-Tumor Immunotherapy**. *Cell biochemistry and biophysics* 2015, **73**(3):723-729.
13. Berg M, Lundqvist A, McCoy P, Jr., Samsel L, Fan Y, Tawab A, Childs R: **Clinical-grade ex vivo-expanded human natural killer cells up-regulate activating receptors and death receptor ligands and have enhanced cytolytic activity against tumor cells**. *Cytotherapy* 2009, **11**(3):341-355.
14. Spanholtz J, Tordoir M, Eissens D, Preijers F, van der Meer A, Joosten I, Schaap N, de Witte TM, Dolstra H: **High log-scale expansion of functional human natural killer cells from umbilical cord blood CD34-positive cells for adoptive cancer immunotherapy**. *PloS one* 2010, **5**(2):e9221.
15. Spanholtz J, Preijers F, Tordoir M, Trilsbeek C, Paardekooper J, de Witte T, Schaap N, Dolstra H: **Clinical-grade generation of active NK cells from cord blood hematopoietic progenitor cells for immunotherapy using a closed-system culture process**. *PloS one* 2011, **6**(6):e20740.
16. Tam YK, Martinson JA, Doligosa K, Klingemann HG: **Ex vivo expansion of the highly cytotoxic human natural killer-92 cell-line under current good manufacturing practice conditions for clinical adoptive cellular immunotherapy**. *Cytotherapy* 2003, **5**(3):259-272.
17. Fujisaki H, Kakuda H, Imai C, Mullighan CG, Campana D: **Replicative potential of human natural killer cells**. *British journal of haematology* 2009, **145**(5):606-613.
18. Chang YH, Connolly J, Shimasaki N, Mimura K, Kono K, Campana D: **A chimeric receptor with NKG2D specificity enhances natural killer cell activation and killing of tumor cells**. *Cancer research* 2013, **73**(6):1777-1786.

Fig. S1A

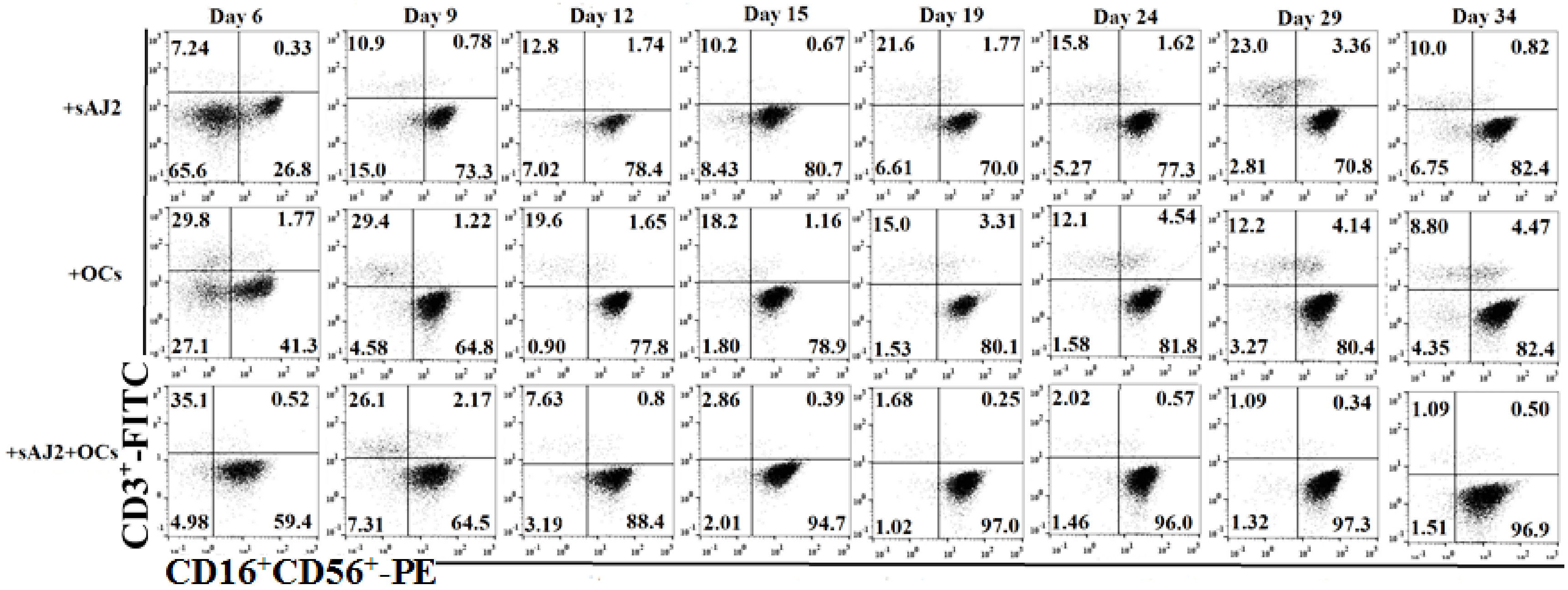

Day 6
Day 9
Day 12
Day 15
Day 19
Day 24
Day 29
Day 34
+sAJ2
+OCs
+sAJ2+OCs
7.24
0.33
65.6
26.8
10.9
0.78
15.0
73.3
12.8
1.74
7.02
78.4
10.2
0.67
8.43
80.7
21.6
1.77
6.61
70.0
15.8
1.62
5.27
77.3
23.0
3.36
2.81
70.8
10.0
0.82
6.75
82.4
29.8
1.77
27.1
41.3
29.4
1.22
4.58
64.8
19.6
1.65
0.90
77.8
18.2
1.16
1.80
78.9
15.0
3.31
1.53
80.1
12.1
4.54
1.58
81.8
12.2
4.14
3.27
80.4
8.80
4.47
4.35
82.4
35.1
0.52
4.98
59.4
26.1
2.17
7.31
64.5
7.63
0.8
3.19
88.4
2.86
0.39
2.01
94.7
1.68
0.25
1.02
97.0
2.02
0.57
1.46
96.0
1.09
0.34
1.32
97.3
1.09
0.50
1.51
96.9
CD3+-FITC
CD16+CD56+-PE

Fig. S1B

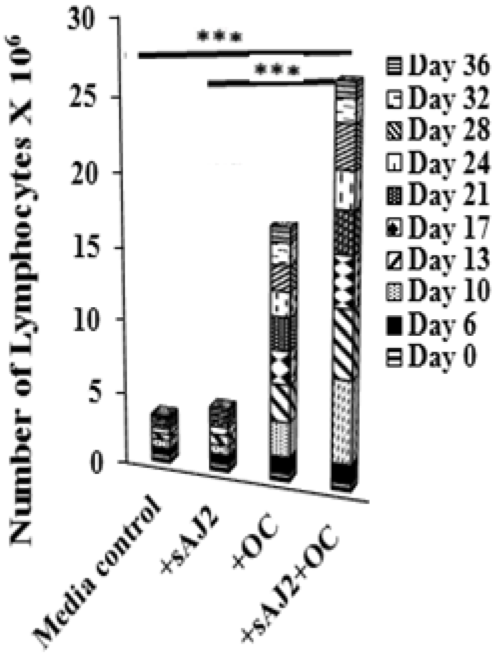

Fig. S1C

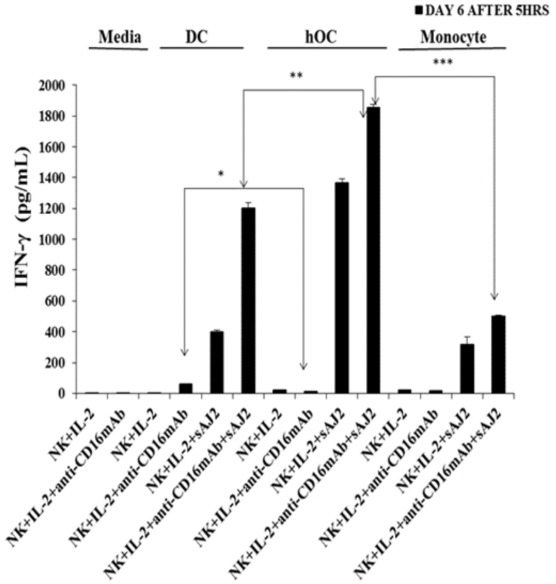

Fig. S1D

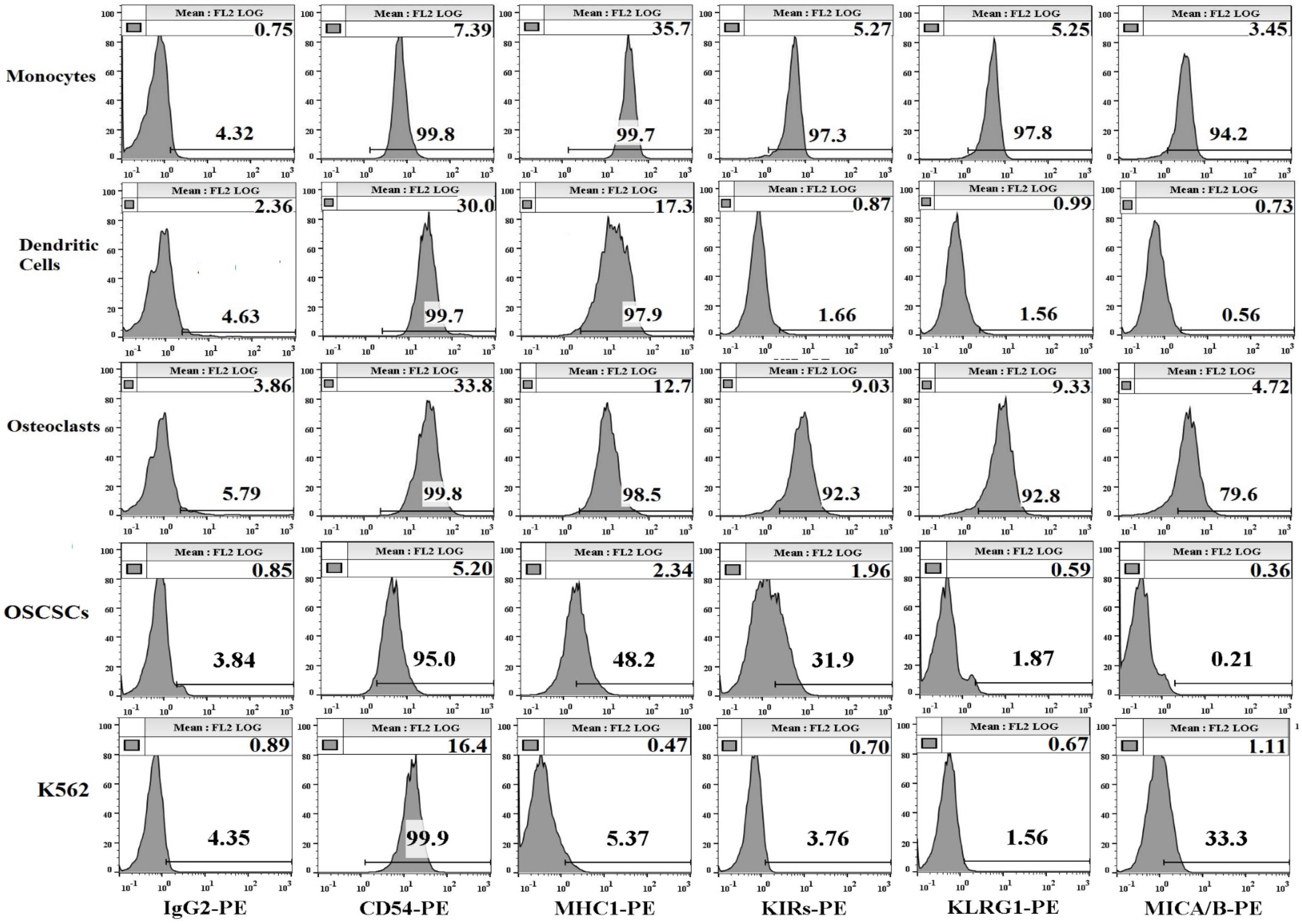
Monocytes
Dendritic Cells
Osteoclasts
OSCSCs
K562
Mean : FL2 LOG
0.75
4.32
7.39
99.8
35.7
99.7
5.27
97.3
5.25
97.8
3.45
94.2
2.36
4.63
30.0
99.7
17.3
97.9
0.87
1.66
0.99
1.56
0.73
0.56
3.86
5.79
33.8
99.8
12.7
98.5
9.03
92.3
9.33
92.8
4.72
79.6
0.85
3.84
5.20
95.0
2.34
48.2
1.96
31.9
0.59
1.87
0.36
0.21
0.89
4.35
16.4
99.9
0.47
5.37
0.70
3.76
0.67
1.56
1.11
33.3
IgG2-PE
CD54-PE
MHC1-PE
KIRs-PE
KLRG1-PE
MICA/B-PE

Fig. S1: Higher expression of NK activating ligands by Osteoclasts.

To generate osteoclasts (OCs), monocytes were cultured in medium containing M-CSF (25 ng/ml) and RANKL (25 ng/ml) for 21 days. Highly purified NK cells ($1x10^6$ cells/ml) were treated with the combination of IL-2 (1000 U/ml) and anti-CD16mAb (3ug/ml) for 18 hours before they were co-cultured with autologous OCs in the presence or absence of sAJ2 bacteria at 1:2:4 ratios (OCs:NK:sAJ2), respectively. Surface expression of CD3, CD16, and CD56 was analyzed in $1x10^4$ lymphocyte samples from co-cultures at days 6, 9, 12, 15, 19, 24, 29, and 34 using flow cytometry, and culture medium was refreshed and supplemented with rh-IL-2 (1000 U/ml) **(A).** Cells were co-cultured as described in Fig. S1A and expanded lymphocytes were counted manually using microscope **(B).** Monocytes were isolated from the PBMCs of a healthy donor. To generate dendritic cells, monocytes were cultured in medium containing GM-CSF (150 ng/ml) and IL-4 (50 ng/ml) for 8 days. Osteoclasts were generated and NK cells were purified as described in Fig. S1A, before they were co-cultured with autologous cells in the presence of sAJ2 bacteria at 1:2:4 ratios (target cells:NK:sAJ2), respectively. On day 6 of the culture, the culture media was removed and the NK cells were treated with rh-IL-2 for 5 hours before the supernatants were harvested, and IFN-γ secretions were determined using single ELISA **(C)**. Monocytes were isolated, dendritic cells and osteoclasts were generated as described in Figs. S1A and D, OSCSCs and K562 tumor cell lines were cultured as described in materials and methods, $1x10^4$ cells were used to analyze MHC-1, CD54, KIR2, KIR3, KLRG1 and MICA/B surface expressions, employing PE-conjugated antibodies and flow cytometry. IgG2 isotype was used as a control **(D).**

Fig. S2A

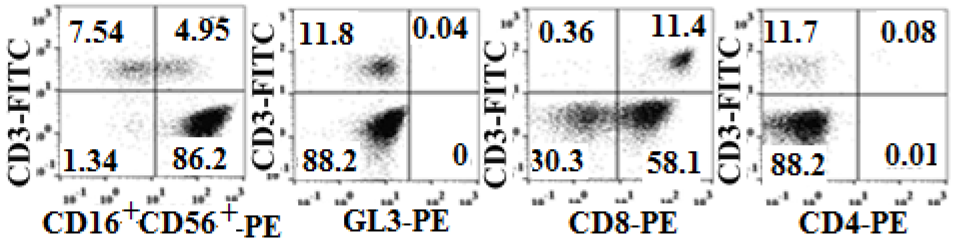

Fig. S2B

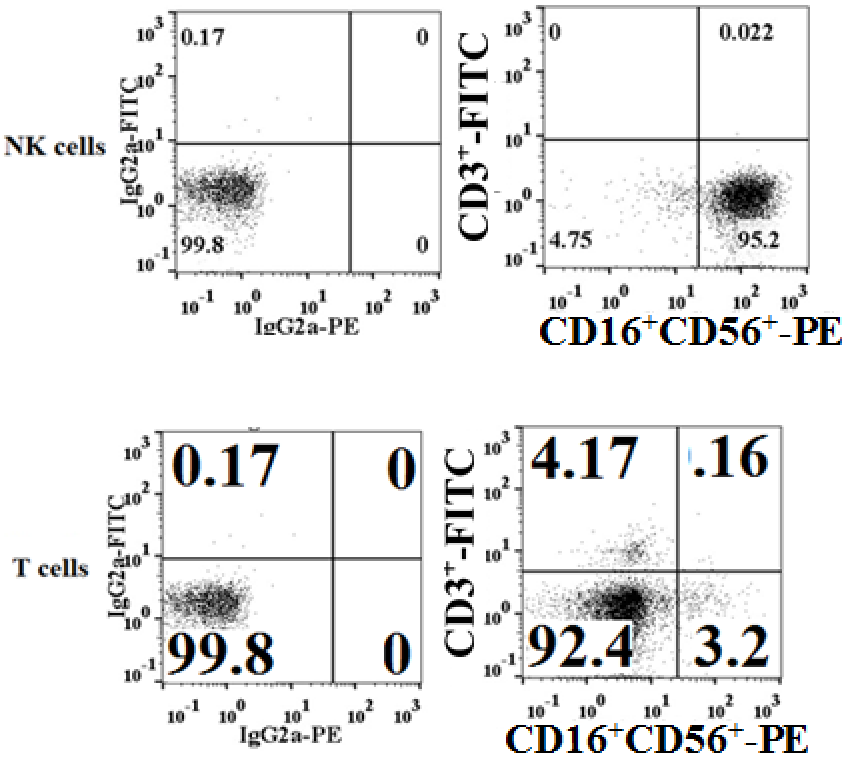

Fig. S2C

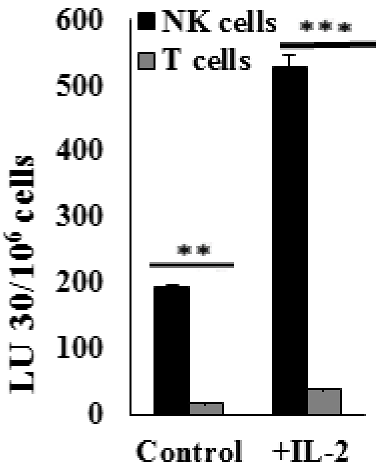

Fig. S2D

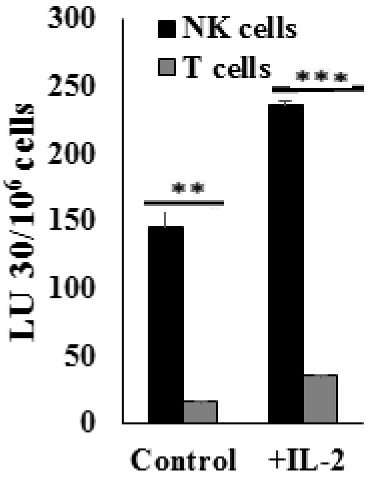

Fig. S2E

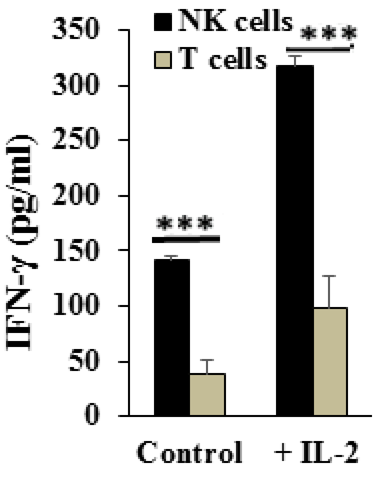

Fig. S2F

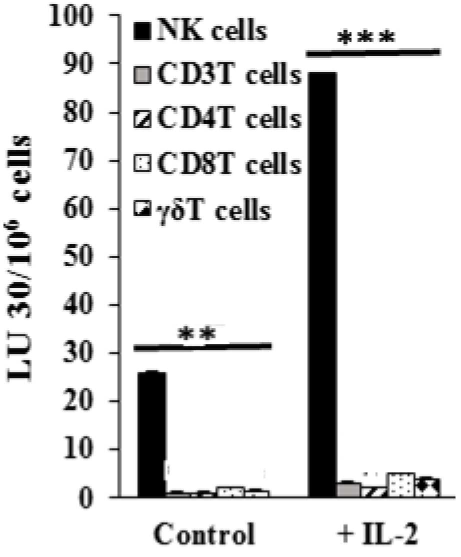

Fig. S2G

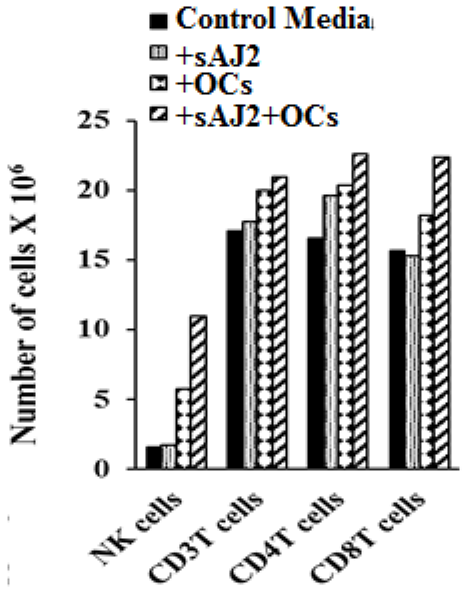

Fig. S2H

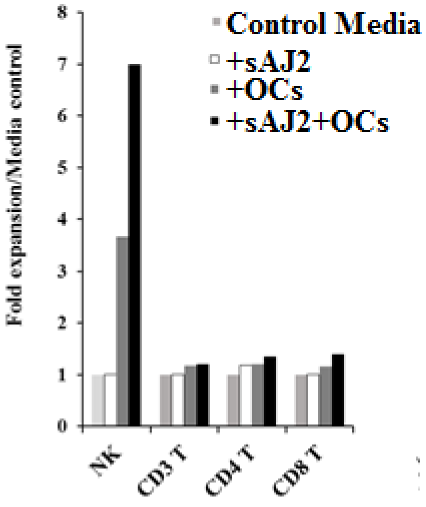

Fig. S2. Unlike NK cells, T cells purified from osteoclast-expanded NK cells do not mediate cytotoxicity against OSCSCs and secrete IFN-γ moderately.

Freshly purified NK cells were treated and co-cultured with monocyte-derived autologous osteoclasts as described in Materials and Methods. Surface expression of CD3, CD16, CD56, GL3 (TCR γ/δ), CD4 and CD8 was analyzed in lymphocyte samples from co-cultures at day 9 using FITC- and PE-conjugated antibodies and flow cytometry **(A)**. NK cells were treated and co-cultured with autologous osteoclasts as described in Fig. S1A and on day 9, CD3T-positive cells were sorted out using CD3T positive selection kit, purity of CD3T-negative (NK) cells was assessed using CD3, CD16, CD56 FITC and PE- conjugated antibodies and flow cytometry **(B).** CD3T-positive cells and CD3T-negative cells (CD16 positive cells) were treated with rh-IL-2 (1000 U/ml) for 18-20 hours before they were tested for cytotoxicity using a standard 4-hour ^{51}Cr release assay against the OSCSCs **(C)** and K562 (**D**) cell lines. The lytic units $30/10^6$ cells were determined using the method described in Materials and Methods, for OSCSCs and K562 respectively. The supernatant was harvested from the culture and IFN-γ secretion was determined using single ELISA **(E).** NK cells, CD3T, CD4T, CD8T, and γδT cells were purified from PBMC as described in materials and methods, and were activated with rh-IL-2 for 18-20 hours, before they were tested for cytotoxicity using a standard 4-hour ^{51}Cr release assay against the OSCSCs **(F).** The lytic units $30/10^6$ cells were determined using the inverse number of lymphocytes required to lyse 30% of OSCSCs x 100 **(F)**. NK and T cells were purified from PBMCs as described in materials and methods, NK cells were treated as described in Materials and Methods. T cells were activated with anti-CD3 (1 ug/ml) and anti-CD28 (3 ug/ml) 18-20 hours before they were cultured with autologous OCs, and expanded lymphocytes were counted manually using microscope day 4 after the culture **(G)**. NK and T cells were purified and cultured with OCs, and counted on day 4 as described in Fig. S2G, fold expansion of

lymphocytes expanded by the OCs were divided by fold expansion of lymphocytes without the OCs **(H)**.

Fig. S3A

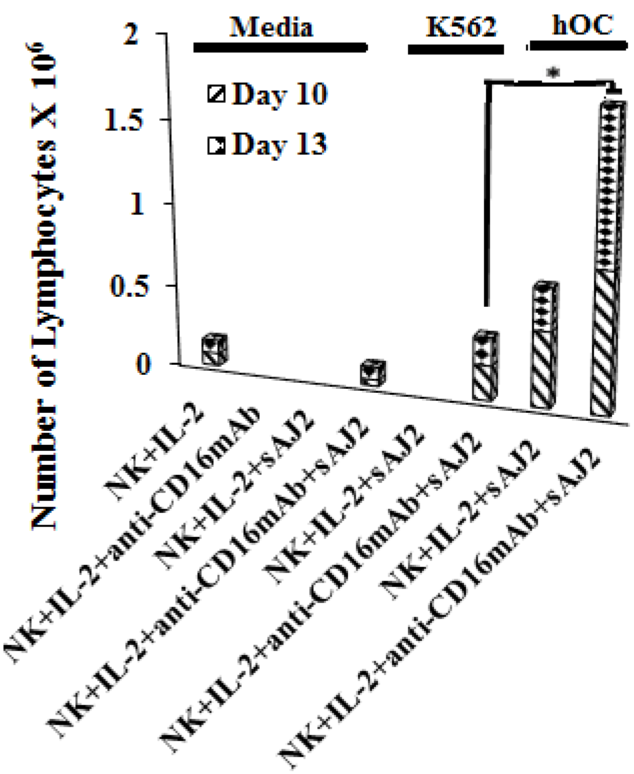

Fig. S3B

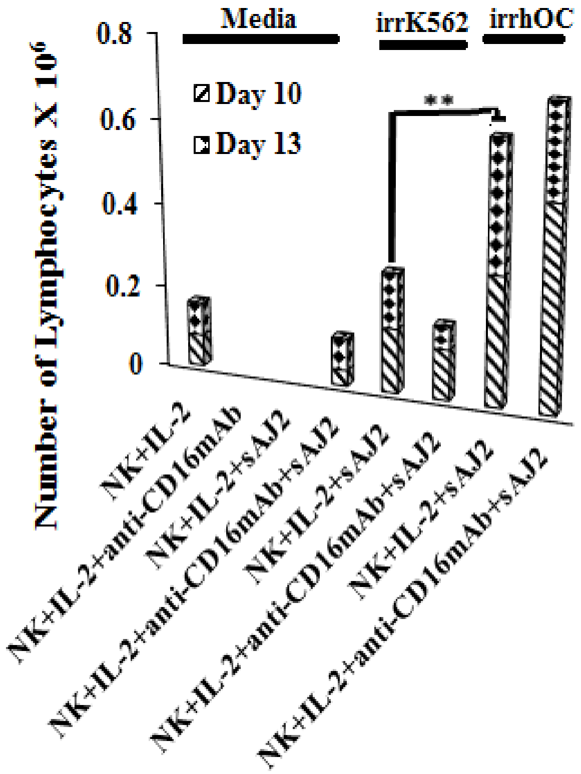

Fig. S3C

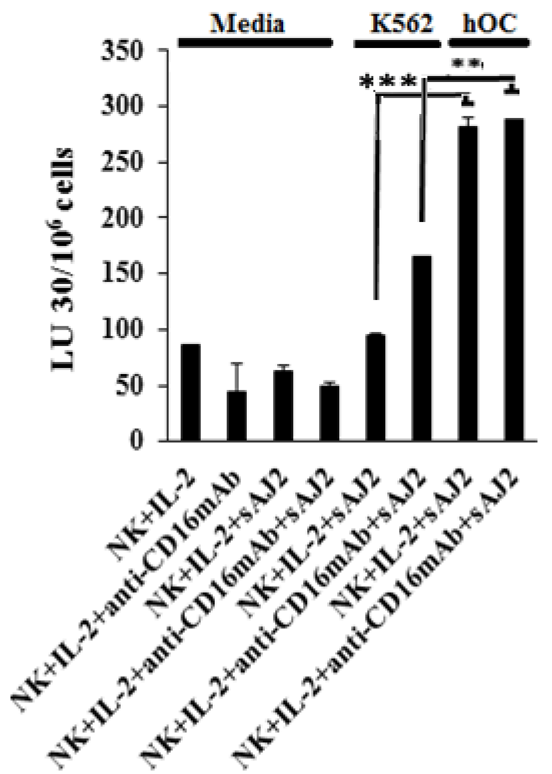

Fig. S3D

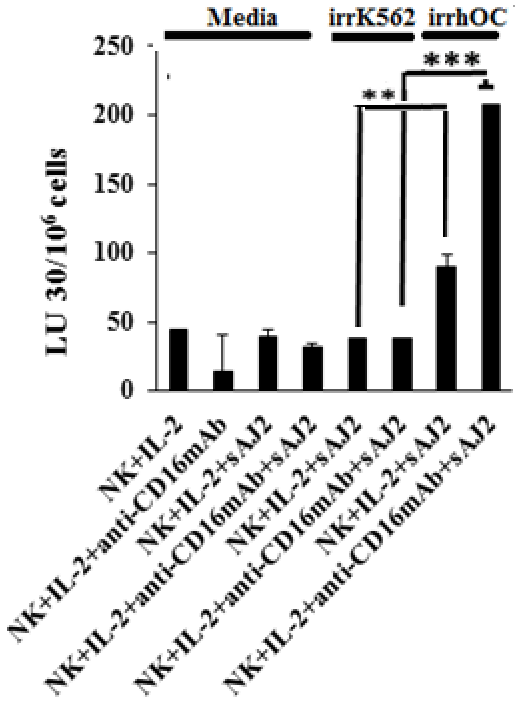

Fig. S3E

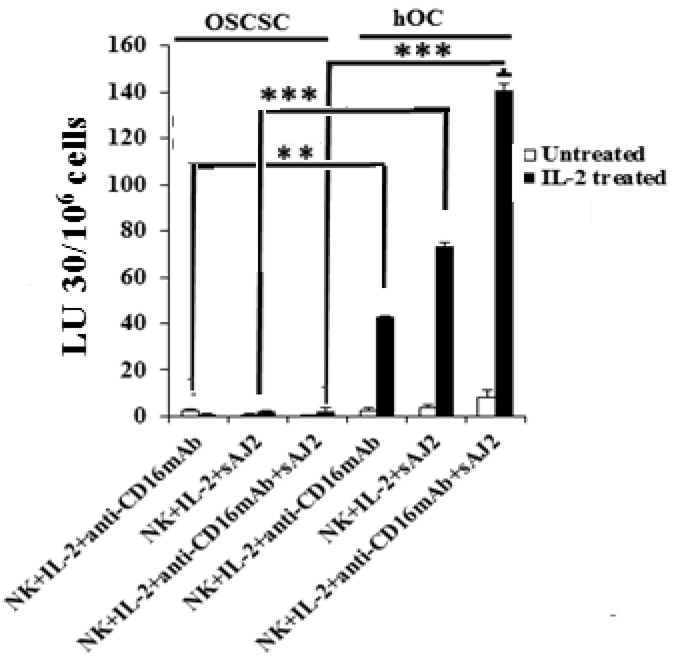

Fig. S3F

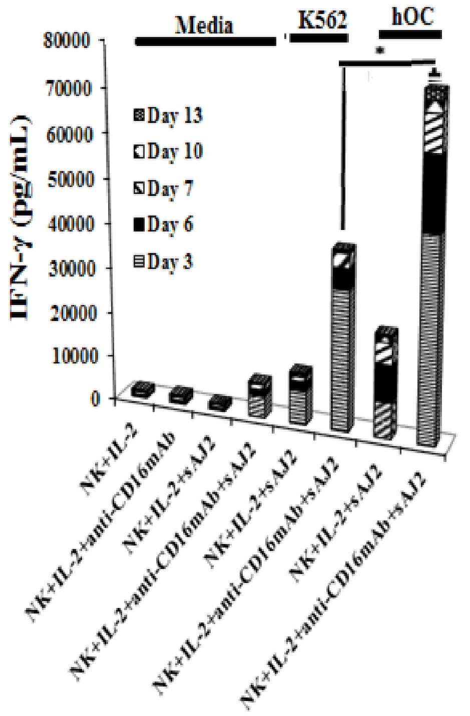

Fig. S3G

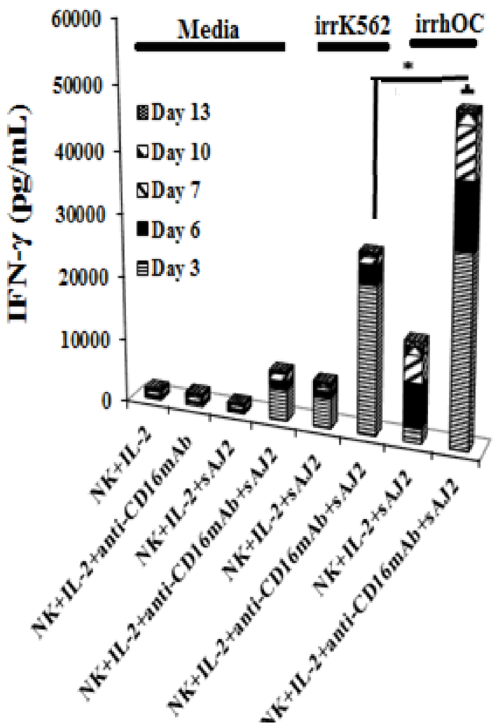

Fig. S3H

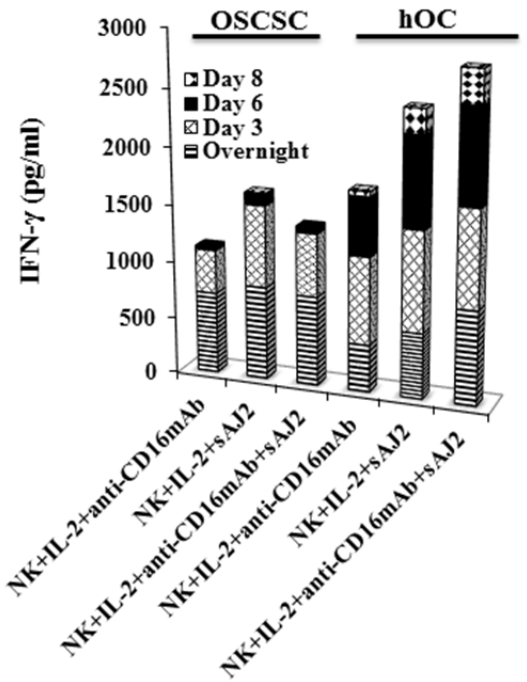

Fig. S3 Osteoclasts, but not K562 or OSCSCs, expand NK cells and increase NK cell function substantially.

To generate osteoclasts, monocytes were cultured in medium containing M-CSF (25 ng/ml) and RANKL (25 ng/ml) for 21 days, K562 tumor cell lines were cultured as described in materials and methods. Highly purified NK cells ($1x10^6$ cells/ml) were treated with the combination of IL-2 (1000 U/ml) and anti-CD16mAb (3ug/ml) for 18 hours before they were co-cultured with K562 and autologous OCs in the presence of sAJ2 bacteria at 1:2:4 ratios (OCs:NK:sAJ2), respectively. Cells from the cultured were counted manually using microscope on day 6, 10 and 13 **(A)**. The osteoclasts generated as described in Fig. S1A and K562 tumor cells lines were irradiated at 40 grays (Gy) as described in the materials and methods. NK cells were purified and treated as described in Materials and Methods, before they were co-cultured with irradiated K562 and irradiated autologous OCs in the presence of sAJ2 bacteria at 1:2:4 ratios (OCs:NK:sAJ2), respectively. Cells from the cultured were counted manually using microscope on day 6, 10 and 13 **(B)**. NK cells were purified and cultured with OCs and K562 as described in Fig. S1A, cytotoxicity of lymphocytes co-cultured for 6 days was determined using a standard 4-hour ^{51}Cr release assay against OSCSCs. The lytic units $30/10^6$ cells were determined using method described in Fig. S2F **(C)**. NK cells were purified and cultured with OCs and K562 as described in Fig. S3B, cytotoxicity of lymphocytes co-cultured for 6 days was determined using a standard 4-hour ^{51}Cr release assay against OSCSCs. The lytic units $30/10^6$ cells were determined using method described in Fig. S2F **(D)**. NK cells were purified and cultured with OCs and OSCSCs as described in Fig. S3A, cytotoxicity of lymphocytes co-cultured for 6 days was determined using a standard 4-hour ^{51}Cr release assay against OSCSCs. The lytic units $30/10^6$ cells were determined using method described in Fig. S2F **(E)**. NK cells were purified and cultured with OCs and K562 as described in Fig. S2A,

supernatant was harvested on day 3, 6, 7, 10 and 13, and IFN-γ secretion was determined using single ELISA (F). NK cells were purified and cultured with irradiated OCs and irradiated K562, as described in Fig. S3B, supernatant was harvested on day 3, 6, 7, 10 and 13, and IFN-γ secretion was determined using single ELISA (G). NK cells were purified and cultured with OCs and OSCSCs as described in Fig. S3A, supernatant was harvested on day 1, 3, 6 and 8, and IFN-γ secretion was determined using single ELISA (H).

Fig. S4A

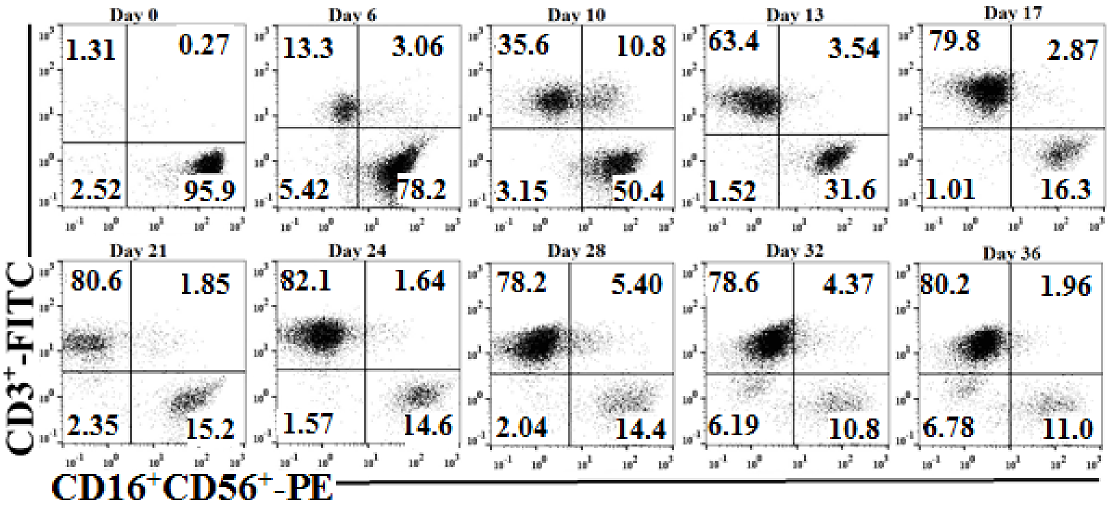

Fig. S4B

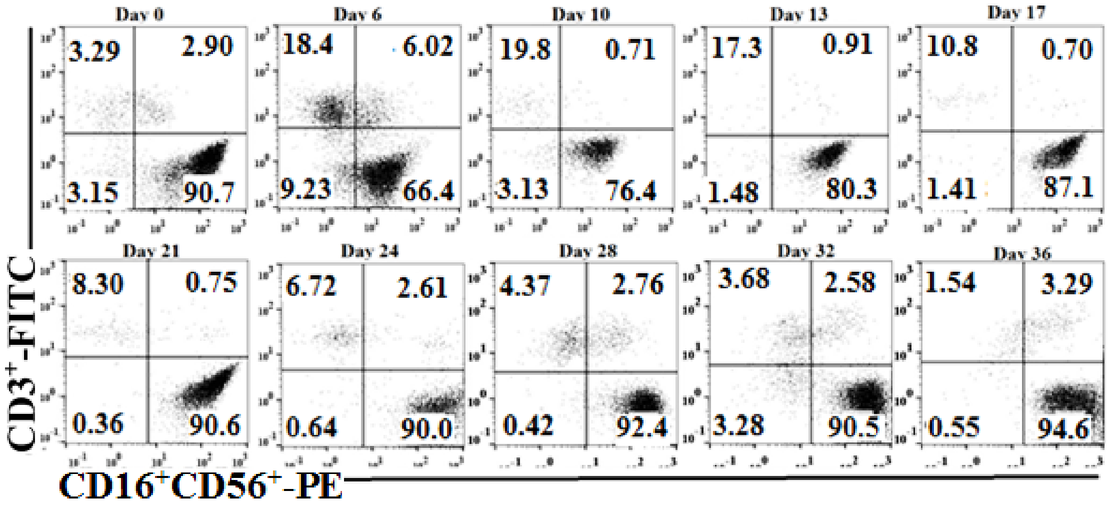

Fig. S4C

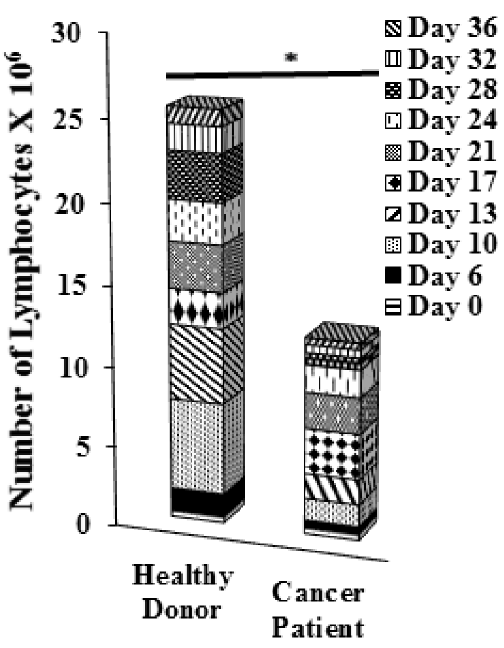

Fig, S4D

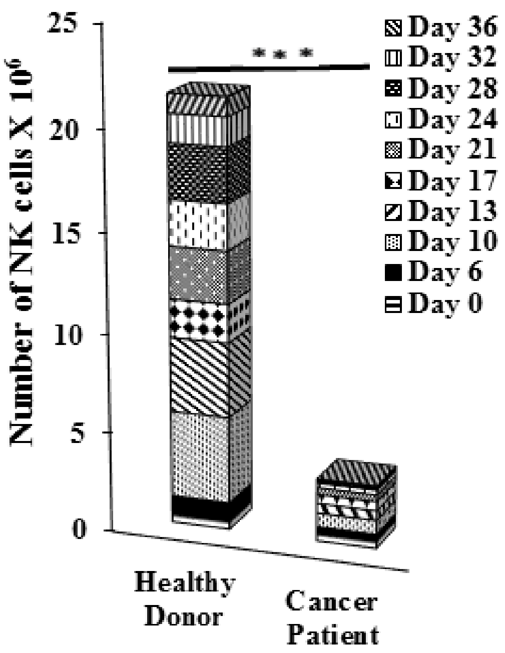

Fig. S4E

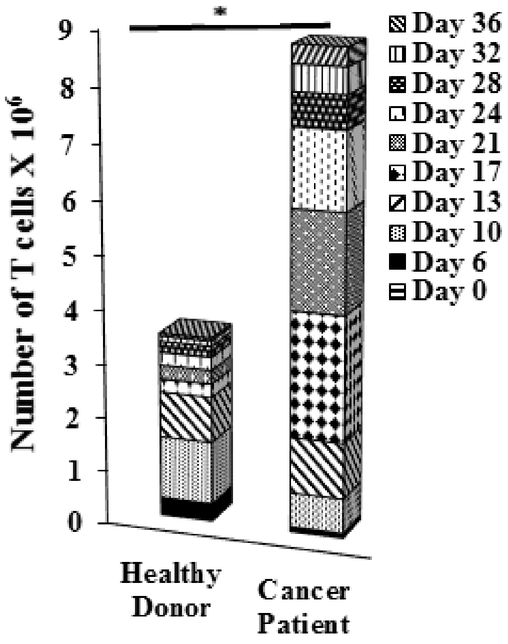

Fig. S4F

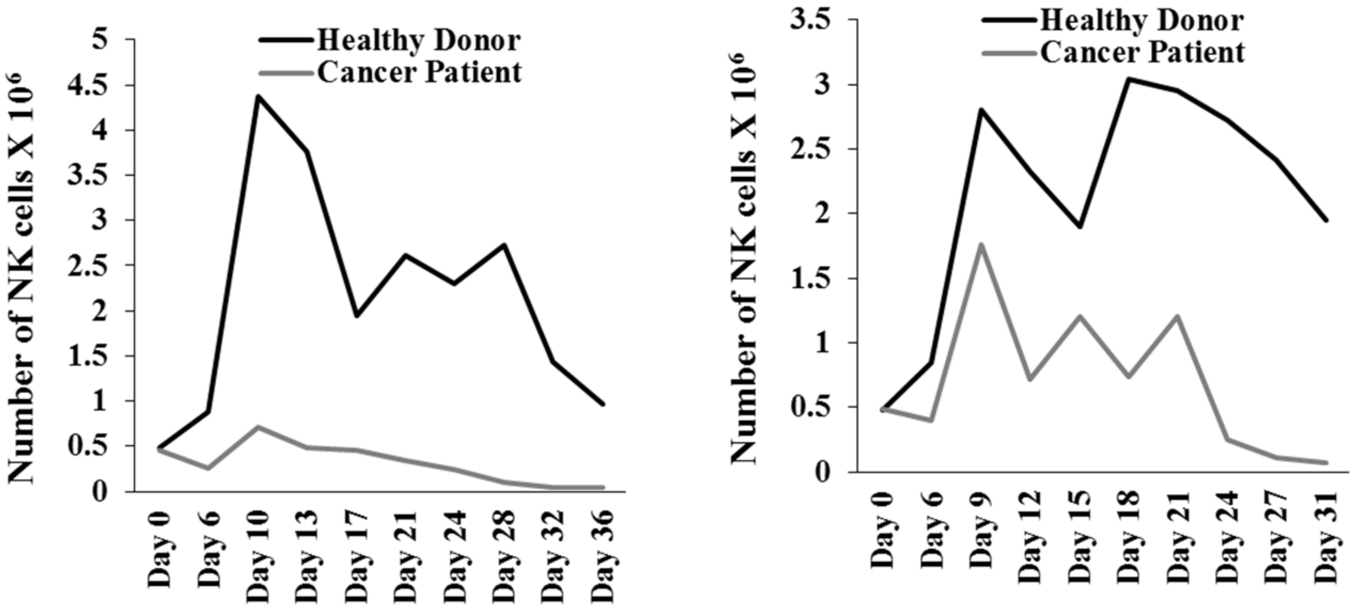
Healthy Donor
Cancer Patient
Number of NK cells X 10^6
5
4.5
4
3.5
3
2.5
2
1.5
1
0.5
0
Day 0
Day 6
Day 10
Day 13
Day 17
Day 21
Day 24
Day 28
Day 32
Day 36
Healthy Donor
Cancer Patient
Number of NK cells X 10^6
3.5
3
2.5
2
1.5
1
0.5
0
Day 0
Day 6
Day 9
Day 12
Day 15
Day 18
Day 21
Day 24
Day 27
Day 31

Fig. S4G

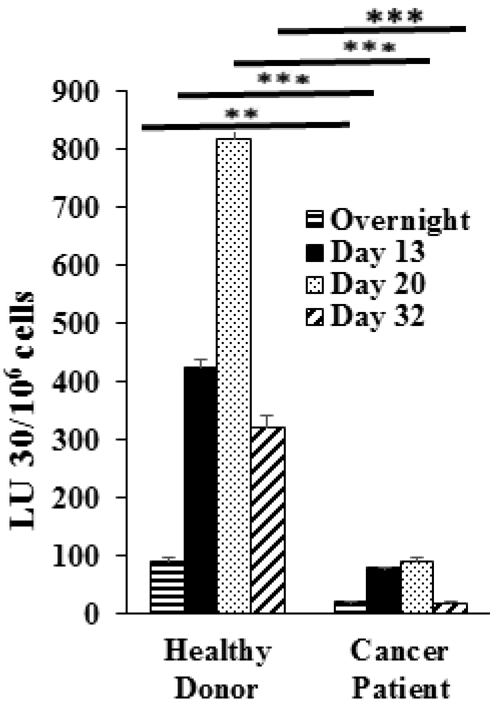

Fig. S4H

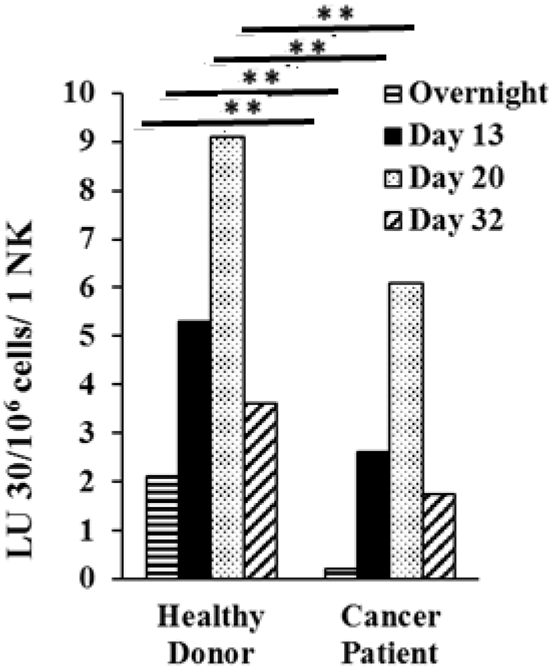

Fig. S4I

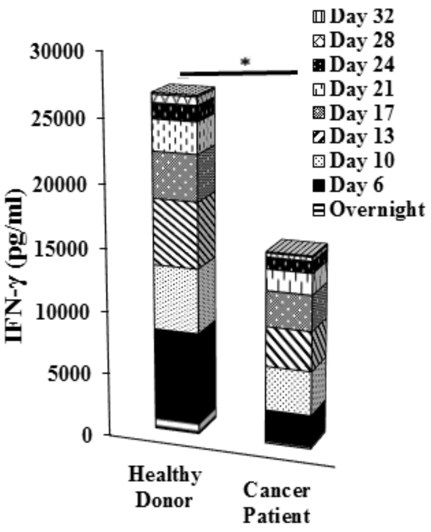

Fig. S4J

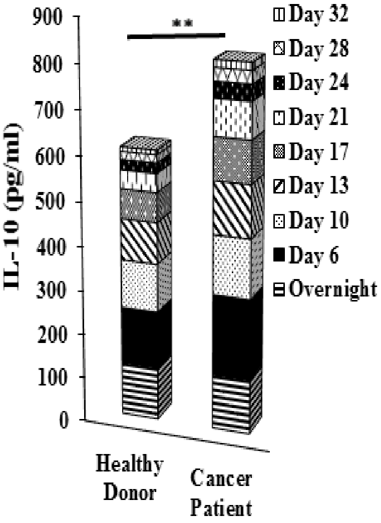

Fig. S4K

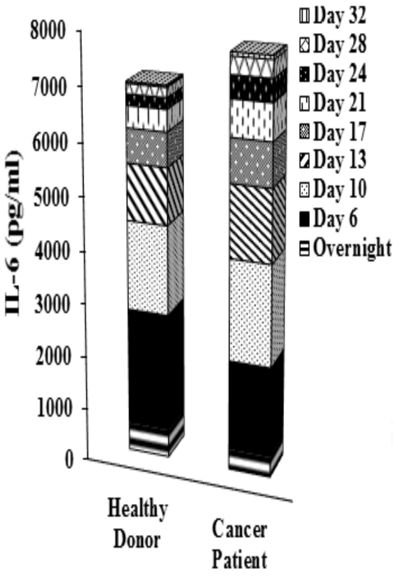

Fig. S4. Small fraction of contaminating T cells within purified NK cells from cancer patient expand faster and crowd out NK cells likely due to decreased NK cell function.

Freshly purified NK cells from a healthy donor and a pancreatic cancer patient were treated and co-cultured with monocyte-derived allogeneic (from different healthy donor) osteoclasts as described in Fig. S1A. Surface expression of CD3, CD16 and CD56 was analyzed in $1x10^4$ lymphocyte samples from co-cultures at days 6, 10, 13, 17, 21, 24, 28, 32 and 36 of cancer patient (**A**) and healthy donor (**B**) using FITC- and PE-conjugated antibodies and flow cytometry. After 6, 10, 13, 17, 21, 24, 28 and 32 days of co-culture, expanded lymphocytes were counted manually using microscope **(C).** Cells counted as mentioned in Fig. S4C and were adjusted based on the surface expression analyzed in Fig. S4A and S4B to determine the number T/NKT cells **(D)** and NK cells at each day **(E and F).** Cytotoxicity of lymphocytes co-cultured for 18-20 hours, 13, 20 and 32 days was determined using a standard 4-hour ^{51}Cr release assay against OSCSCs. The lytic units $30/10^6$ cells were determined using method described in Fig. S2F **(F)**. Lytic units from Fig. S4F was adjusted based on surface expression analyzed is Fig. S4A and S4B to determine the cytotoxicity mediated by 1 NK cell against OSCSCs **(G)**. The supernatant was harvested from the overnight, 6, 10, 13, 17, 21, 24, 28 and 32 days of co-culture and IFN-γ **(H)**, IL-10 **(I)**, and IL-6 **(J)** secretion was determined using single ELISA.

Fig. S5A

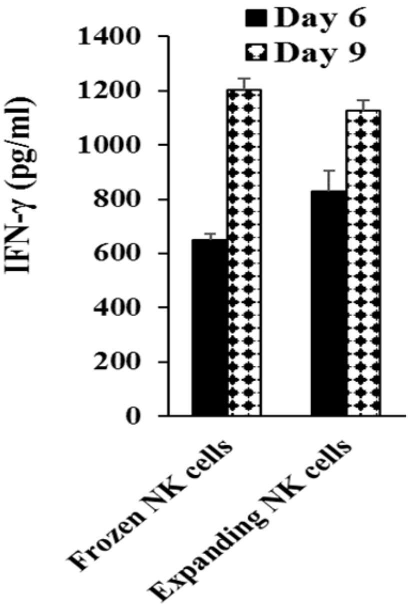

Fig. S5B

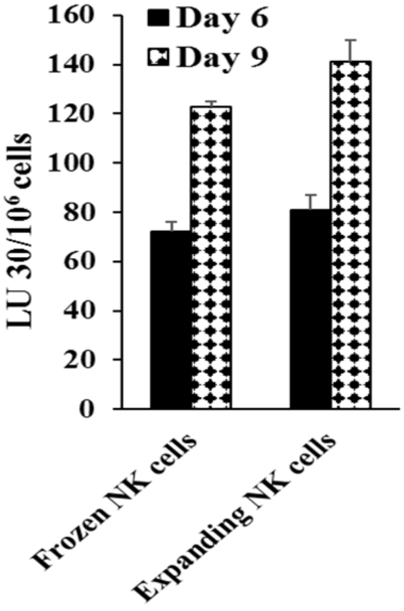

Fig. S5. Osteoclast-expanded NK cells retain their cytokine secretion and cytotoxic function after freezing.

Freshly purified NK cells were treated and co-cultured with monocyte-derived autologous osteoclasts as described in Fig. S1A, day 9 after the cultures, expanded NK cells were frozen. NK cells were thawed and treated with rh-IL-2 (1000 U/ml), day 6 and day 9 after the culture, the supernatant was harvested and IFN-γ secretion was determined using single ELISAs **(A)**. NK cells were cultured as described in Fig. S1A, and the cytotoxicity of lymphocytes day 6 and 9 after the culture was determined using a standard 4-hour ^{51}Cr release assay against OSCSCs. The lytic units $30/10^6$ cells were determined using method described in Fig. S2F **(B)**

Fig. S6

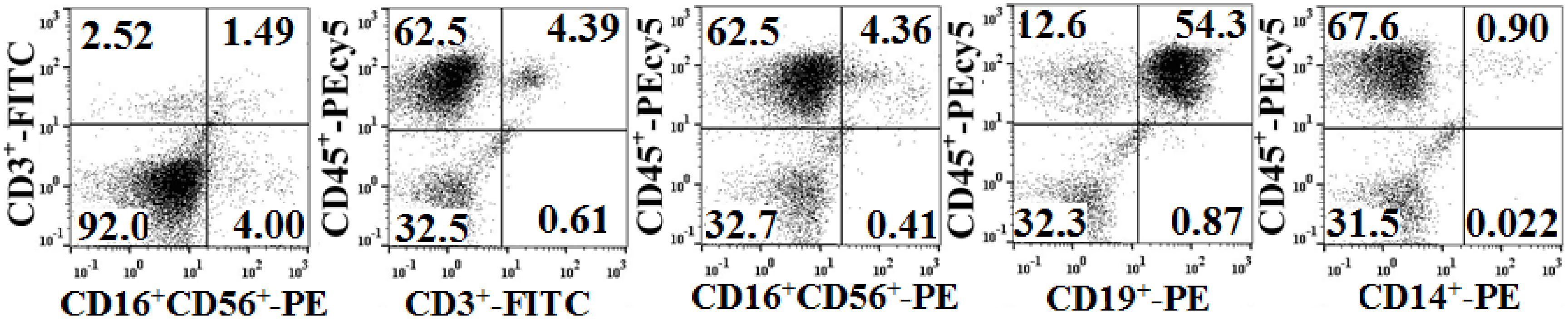

Fig. S6. Phenotype of CD3 T cell depleted lymphocytes from the splenocytes of hu-BLT mice. Humanized-BLT (hu-BLT; human bone marrow/liver/thymus) mice were generated by surgical implantation of human fetal liver and thymus tissue under the renal capsule of 6-8 weeks old immunocompromised NOD.CB17-Prkdcscid/J and NOD.Cg-Prkdcscid Il2rgtm1Wjl/SzJ (NSG) mice. 4-6 weeks post tissue transplant, mice were sub-lethally irradiated and intravenously injected with $CD34^+$ cells isolated from fetal liver to support full reconstitution of human bone marrow. 8-12 weeks after injection with $CD34^+$ cells, reconstitution of human immune system was analyzed using blood specimens. At the end of this experiment engraftment of human immune cells was confirmed by staining splenocytes and bone marrow cells with anti-human CD45, CD3, CD4 and CD8 antibodies and analyzed by flow cytometry (data not shown). Successfully reconstituted hu-BLT mice (levels and lineages of T cells comparable to healthy donors) were orthotopically injected with $1x10^6$ of human OSCSCs into the floor of the mouth. Disease

progression and weight loss was monitored for another 3-4 weeks. Animals were sacrificed, spleens were harvested from the sacrificed animals, and single cell suspensions were obtained as described in materials and methods. CD3T cells were sorted out using human CD3T positive selection kit. Flow through cells (CD3-negative cells) were analyzed for surface expression of human CD3, CD16, CD56, CD45, CD19, CD14, after staining with the respective PE-conjugated, PE-Cy5-conjugated and FITC-conjugated antibodies. Isotype control antibodies were used as a control **(Fig. S6)**.

Fig. S7A

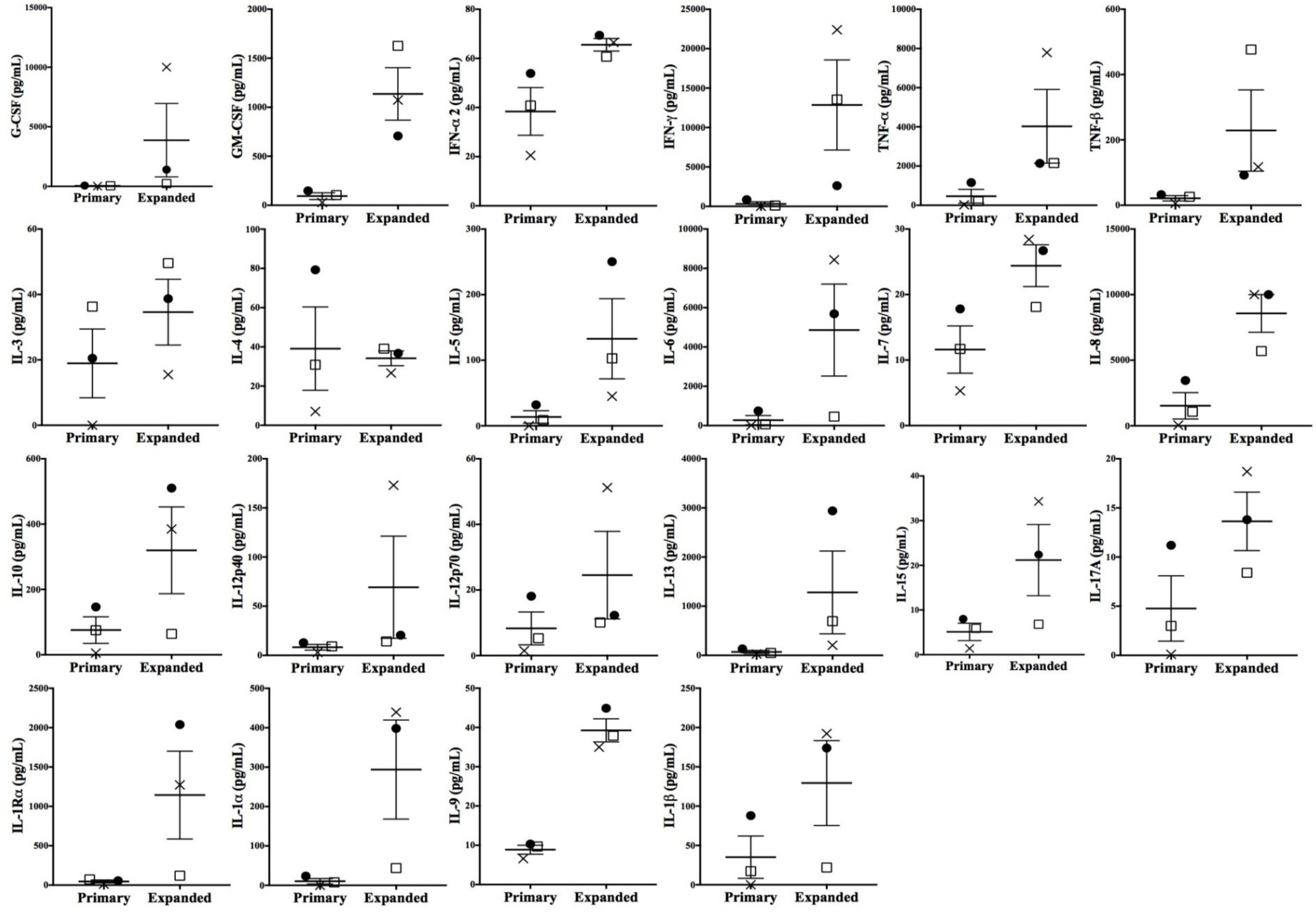

G-CSF (pg/mL)
GM-CSF (pg/mL)
IFN-α 2 (pg/mL)
IFN-γ (pg/mL)
TNF-α (pg/mL)
TNF-β (pg/mL)
IL-3 (pg/mL)
IL-4 (pg/mL)
IL-5 (pg/mL)
IL-6 (pg/mL)
IL-7 (pg/mL)
IL-8 (pg/mL)
IL-10 (pg/mL)
IL-12p40 (pg/mL)
IL-12p70 (pg/mL)
IL-13 (pg/mL)
IL-15 (pg/mL)
IL-17A (pg/mL)
IL-1Rα (pg/mL)
IL-1α (pg/mL)
IL-9 (pg/mL)
IL-1β (pg/mL)
Primary
Expanded

Fig. S7B

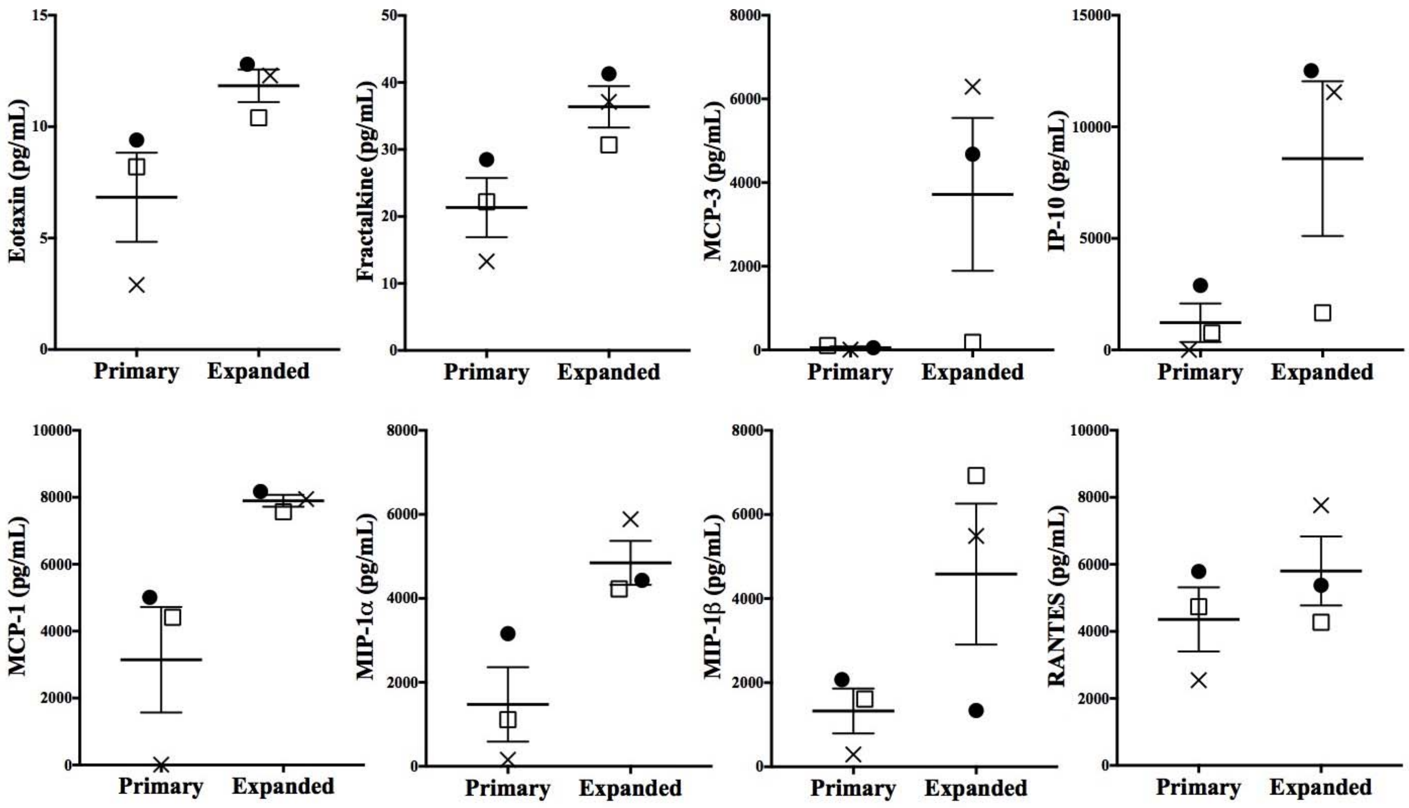
Eotaxin (pg/mL)
Fractalkine (pg/mL)
MCP-3 (pg/mL)
IP-10 (pg/mL)
MCP-1 (pg/mL)
MIP-1α (pg/mL)
MIP-1β (pg/mL)
RANTES (pg/mL)
Primary
Expanded

Fig. S7C

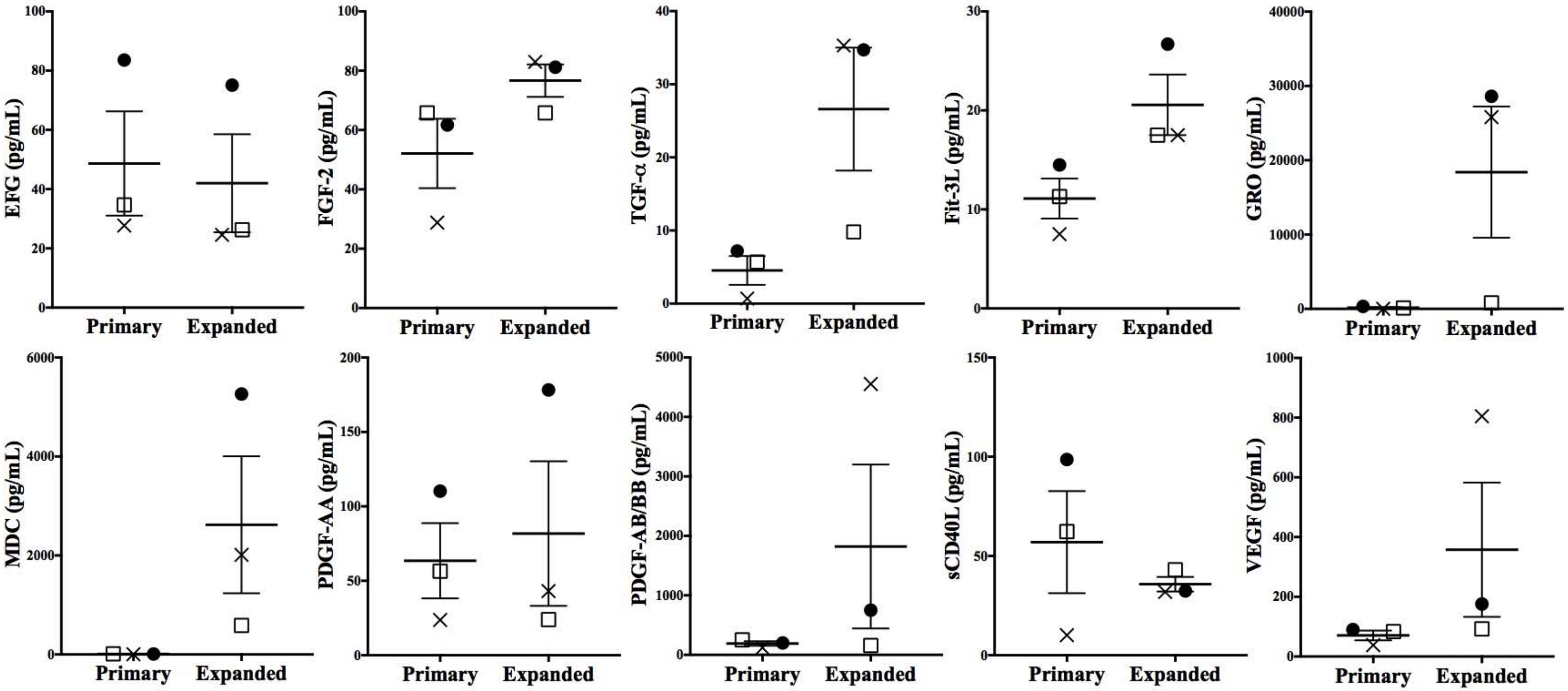

Fig. S7. Cytokines, chemokines and growth factors and ligands secreted by primary and osteoclast-expanded NK cells

Highly purified NK cells and monocytes were obtained from peripheral blood mononuclear cells (PBMCs) of healthy donors and NK cells were treated ($1x10^6$ cells/ml) with IL-2 (1000 U/ml) for 18 hours before the supernatant was harvested. To generate osteoclasts, monocytes were cultured in alpha-MEM media containing M-CSF (25 ng/ml) and RANKL (25 ng/ml) for 21 days. For expansion,

purified NK cells (1x10^{6} cells/ml) were treated with the combination of IL-2 (1000 U/ml) and anti-CD16mAb (3ug/ml) for 18 hours before they were co-cultured with autologous osteoclasts in the presence of sAJ2 bacteria at 1:2:4 ratios (OC:NK:sAJ2), respectively. The supernatant was harvested after 6 days of co-culture and multiplex assay was used to determine cytokines **(A)**, chemokines **(B)** and growth factors **(C)** levels secreted by the primary and expanded NK cells.

Fig. S8

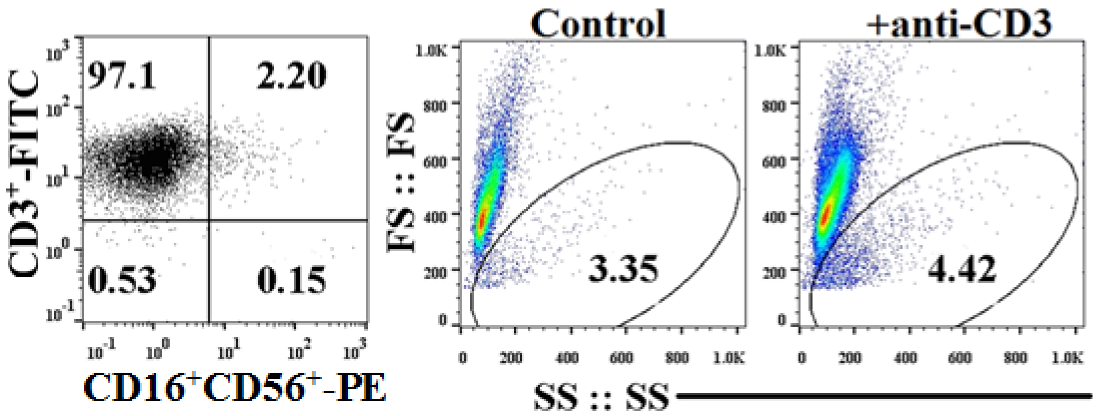

Fig. S8. Purified T cells treated with anti-CD3mAb in the absence of NK cells did not lose forward and side scatter.

Highly purified T cells and monocytes were obtained from peripheral blood mononuclear cells (PBMCs) of healthy donors and T cells were treated ($1x10^6$ cells/ml) with IL-2 (100 U/ml) and anti-CD3 (1ug/ml) for 18 hours before they were co-cultured with autologous osteoclasts in the presence of sAJ2 bacteria at 1:2:4 ratios (OC:T cells:sAJ2), respectively. The cells were analyzed for CD3, CD16 and CD56 on day 9 after the culture. Anti-CD3 treated T cells did not lose Forward and side scatter in the absence of NK cells **(Fig. S8)**.
